# Independent and Interactive Associations of Fitness and Fatness With Changes in Cardiometabolic Risk in Children: A Longitudinal Analysis

**DOI:** 10.3389/fendo.2020.00342

**Published:** 2020-06-12

**Authors:** Xianwen Shang, Yanping Li, Haiquan Xu, Qian Zhang, Xiaoqi Hu, Ailing Liu, Songming Du, Tingyu Li, Hongwei Guo, Ying Li, Guifa Xu, Weijia Liu, Jun Ma, Guansheng Ma

**Affiliations:** ^1^Chinese Center for Disease Control and Prevention, National Institute for Nutrition and Health, Beijing, China; ^2^School of Behavioural and Health Sciences, Australian Catholic University, Fitzroy, VIC, Australia; ^3^Faculty of Medicine, Dentistry and Health Sciences, University of Melbourne, Melbourne, VIC, Australia; ^4^Department of Nutrition, Harvard T. H. Chan School of Public Health, Boston, MA, United States; ^5^Institute of Food and Nutrition Development, Ministry of Agriculture and Rural Affairs, Beijing, China; ^6^Department of Pediatrics, Chongqing Children's Hospital, Chongqing, China; ^7^School of Public Health, Fudan University, Shanghai, China; ^8^Department of Nutrition and Food Hygiene, Public Health College, Harbin Medical University, Harbin, China; ^9^Department of Public Health, Shandong University, Jinan, China; ^10^School Health Department, Guangzhou Center for Disease Control and Prevention, Guangzhou, China; ^11^Institute of Child and Adolescent Health, School of Public Health, Peking University, Beijing, China; ^12^Department of Nutrition and Food Hygiene, School of Public Health, Peking University, Beijing, China

**Keywords:** cardiorespiratory fitness, fatness, cardiometabolic risk, mediation analysis, moderation analysis

## Abstract

**Background:** Findings for associations between cardiorespiratory fitness (CRF) and cardiometabolic risk (CMR) factors are inconsistent, and the interactive association between CRF and fatness with CMR factors is unclear in children. Our study aimed to examine whether CRF and fatness are independently and interactively associated with CMR factors.

**Methods:** We included 5,869 children aged 6–13 years in the analysis. Physical examinations, blood tests, and CRF were measured at baseline and 1 year later. Cardiometabolic risk score (CMRS) was computed by summing *Z* scores of waist circumference (WC), averaged systolic and diastolic blood pressure, glucose, high-density lipoprotein cholesterol (HDL-C, multiplied by −1), and triglycerides.

**Results:** There was a high correlation between fatness and CRF in both boys and girls. High baseline CRF was independently associated with favorable changes in CMRS, BMI, WC, percent body fat (PBF), total cholesterol, LDL-C, and HDL-C (all *P* < 0.025). Improved CRF was independently associated with favorable changes in CMRS, BMI, WC, PBF, total cholesterol, LDL-C, HDL-C, triglycerides, and fasting glucose (all *P* < 0.0321). Baseline BMI was positively associated with changes in CMRS, WC, blood pressure, triglycerides, insulin, and HOMA-IR (all *P* < 0.0462). Low PBF at baseline was associated with favorable changes in CMRS, BMI, WC, blood pressure, HDL-C, triglycerides, insulin, and HOMA-IR (all *P* < 0.0423). The percentage of the total effect of baseline CRF on changes in CMRS, triglycerides, HDL-C, PBF, and WC mediated by baseline BMI was 66.0, 61.6, 40.3, 20.7, and 9.2%, respectively. Baseline CRF was a significant mediator for the association between baseline BMI and changes in CMRS (mediated by 4.3%), triglycerides (5.1%), and HDL-C (12.0%). An inverse association was found between baseline CRF and CMRS in children with high baseline BMI/PBF only. Improved CRF was associated with decreased BMI and WC in children with low baseline CRF.

**Conclusions:** Fatness and CRF are each independently associated with changes in CMR factors. Fatness is a major mediator for the association between CRF and CMR factors, whereas the association between fatness and CMR factors is also mediated by CRF. The beneficial effect of high CRF on CMR factors was more evident in obese or unfit children.

## Introduction

Cardiometabolic risk (CMR) factors including high levels of body mass index (BMI), glucose, cholesterol, and blood pressure accounted for more than 30.0% of global mortality in 2015 (1). There was a high prevalence of CMR factors in both children and adults in China (2), which may be largely due to the dramatic shift toward high energy-density diets and low physical activity lifestyle in the last two decades (3, 4). While childhood CMR factors are highly likely to persist into adulthood (5, 6), suggesting the importance of prevention of these risk factors at an early stage. Prevention of obesity and improvement in cardiorespiratory fitness (CRF) are thought to play an important role in CMR reduction (7–11).

An increasing body of evidence reveals an obesity paradox in cardiovascular disease (CVD) in adults that there is a better prognosis in overweight and mildly obese CVD patients than their leaner counterparts (12, 13). This obesity paradox has attracted increasing concerns over which of fitness and fatness is more important for the development of CVD (14). Some studies found BMI was a stronger predictor of CMR factors in children (15–18), whereas others showed CRF was an independent predictor for CMR factors regardless of BMI (19, 20). More recent research in adults suggests that CRF and adiposity interplayed on changes in CMR factors (12, 13); however, data from children are limited.

Several recent cross-sectional studies in children from Spain have shown that BMI was a mediator of the association between CRF and CMR factors (21, 22). Another cross-sectional study also from Spain performed moderation analysis and found the inverse association between CRF and CMR was more likely to be manifested in obese children (23). However, these studies are restricted by cross-sectional design or small sample sizes. A longitudinal analysis may provide more convincing evidence on the physiological pathways on the interactive and independent effects of CRF and fatness on CMR factors given its advantages in many aspects of a mediation and moderation model that are unavailable in cross-sectional data (24, 25).

We assumed that fatness than CRF was more predictive of CMR factors, and CRF and fatness were interactively associated with the changes in CMR factors in children. The present study examined whether CRF and fatness measured by BMI and percent body fat (PBF) are independently associated with changes in CMR factors in children. We also examined the association between CRF and changes in CMR factors that are moderated and mediated by fatness and whether the association between fatness and changes in CMR factors is mediated by CRF.

## Materials and Methods

### Study Design

The nutrition-based comprehensive intervention study on childhood obesity in China is a multicenter, randomized cluster controlled trial, and the design has been detailed elsewhere (26). The study was conducted in six capital or province capital cities: Beijing, Harbin, Jinan, Shanghai, Chongqing, and Guangzhou. Children in the intervention group received nutrition lectures (knowledge, attitudes, and dietary habits), as well as participated in two times of 10 min or one time of 20 min of Happy 10 program per day (involves various physical activities such as games, dance, and gymnastics, which were designed to stimulate children to enjoy physical activity). No interventions were conducted in the control group. Our previous work has shown the beneficial effects of the intervention on the changes in obesity makers including BMI and waist circumference (WC) (27). Children with serious diseases such as congenital heart disease and schools who participated or aimed to participate in any other research projects within 1 year were excluded. Data on 50-m × eight-shuttle run (50MESR) were not collected in Beijing such that data from the other five cities were included in our analysis. We excluded participants who were from Beijing (*n* = 2,150), those who were lost to follow-up (*n* = 829), and those who had missing values in CRF or BMI at baseline (*n* = 1,019). We included 5,869 children from 303 classes within 30 schools in the final analysis (Figure S1).

The study protocol was approved by the Ethical Review Committee of the National Institute for Nutrition and Food Safety, Chinese Center for Disease Control and Prevention. Oral assent was collected from children, whereas written informed consent was obtained from the next of kin, carers, or guardians of all participants.

### Physical Examinations and Blood Tests

Physical examinations (fasting 10–14 h beforehand), and blood tests were performed at both baseline (May 2009) and follow-up (May 2010) following standardized procedure by trained staff. Blood samples were taken between 7:30 and 8:30 am, and other examinations were then conducted between 8:45 and 9:30 in the morning.

Height was measured to the nearest 0.1 cm using a freestanding stadiometer (GMCS-I; Xindong Huateng Sports Equipment Co. Ltd., Beijing, China), and weight was measured to the nearest 0.1 kg using a double ruler scale (RGT-140; Wujin Hengqi Co. Ltd., Changzhou, China). Body mass index was computed as weight in kilograms divided by height in meters squared. Overweight was defined using BMI according to the International Obesity Task Force standard (28). Waist circumference was measured twice to the nearest 0.1 cm, and the average was used.

Impedance, phase, resistance, and reactance were measured using a single frequency (50 Hz) hand-to-foot bioelectrical impendence device (ImpDF50; Impedimed Pty Ltd., Queensland, Australia) to estimate the whole-body fat. Besides 10–14 h of fasting, children were required to (1) no water drinking in preceding 4 h, (2) no intensive physical activity in preceding 2 h, (3) urine emptying within 30 min, (4) no alcohol drinking in preceding 12 h, and (1) no period on the testing day for girls. Body fat mass was computed using the prediction formula developed by Deurenberg et al. (29), and PBF was then calculated.

Diastolic and systolic blood pressure (DBP and SBP) were measured in the seated position using a mercury sphygmomanometer by trained nurses. The first and fifth Korotkoff sounds were used to represent the SBP and DBP. Three measurements were taken to the nearest two mm Hg, and the average of the last two measurements was used. The mean arterial pressure (MAP) was calculated as DBP + (0.33 × [SBP – DBP]).

Fasting blood samples (5 mL) were collected in serum separator tubes in the morning after 10–14 h of overnight fast and then transported to be clotted for 20–30 min and centrifuged for 10 to 15 min at 3,200 rpm using a Multifuge 35R. Blood samples for glucose tests were transported to the clinical laboratory in a cooler with an ice block at 4°C. Other blood samples were stored in a deep freezer at −70°C until further testing. Fasting glucose was measured using the glucose-oxidized method (Daiichi Pharmaceutical Co., Ltd., Tokyo, Japan). Fasting insulin was measured using the Microparticle Enzyme Immunoassay method (the AxSYM insulin assay; Abbott Co., Ltd., Tokyo, Japan). The Homeostatic Model Assessment of Insulin Resistance (HOMA-IR) was computed as (fasting insulin [μU/L] × fasting glucose [mg/dL])/405. Conventional enzymatic assays were used to measure triglycerides (TGs), total cholesterol (TC), high-density lipoprotein cholesterol (HDL-C), and low-density lipoprotein cholesterol (LDL-C) with 7080 Automatic Analyzer (Daiichi Pharmaceutical Co., Ltd.). Values of TG, TG to HDL-C ratio, insulin, and HOMA-IR were log-transformed.

### Cardiometabolic Risk Assessment

Cardiometabolic risk score (CMRS) was computed by summing age- and sex-specific *Z* scores of WC, the average of SBP and DBP, fasting glucose, HDL-C (multiplied by −1), and TG (30). Waist circumference was removed from this score in the mediation analysis for CRF and BMI with CMRS given the high correlation between WC and BMI.

### Physical Fitness Measurements

50MESR as a traditional test in China was used to evaluate CRF among children (31). A physical education teacher and researcher were involved in the test. Before the test, they would check if the children were under good health conditions. The test would be terminated if the children were struggling. 50MESR test required children to run back and forth four times at their highest speed along a track between two poles set 50 m apart and to turn around the poles counterclockwise. The test was recorded to the nearest 0.1 s (HS-70W stopwatch; CASIO, Shenzhen, China). To motivate children to try their best in the test, the results would be accounted for as a component of the performance assessment of the physical education for the semester. We used the 50MSR speed (400 m divided by test time in seconds) as CRF with a higher value indicating better performance. Cardiorespiratory fitness was analyzed in quintiles as previous studies classified individuals as fit (≥20th centile) and unfit (<20th centile) using centiles of physical fitness (32, 33).

### Covariates

Information about age, sex, class, and grade was provided by the selected schools and confirmed by the children using an administered questionnaire. Dietary intake was assessed using the 24-h diet recall for 3 consecutive days including 2 weekdays and 1 weekend day. Interviews were conducted by trained investigators. Physical activity was assessed using a validated questionnaire, from which metabolic equivalent (MET) was calculated (34). Puberty status was recorded by investigators during the interview when physical examinations were conducted. Children were considered among puberty if the onset of menstruation for girls and the first ejaculation for boys occurred. Birth weight, household income, parental education, and parental height and weight were reported by the parents using a self-administered questionnaire.

### Statistical Analysis

Continuous variables were expressed as means ± standard deviation (SD) and categorical variables as frequency and percentage for baseline characteristics of participants. The *t*-test for continuous variables and the χ^2^ test for categorical variables were used to examine whether the description of baseline characteristics differed between boys and girls.

Body mass index, WC, PBF, SBP, DBP, MAP, TC, HDL-C, LDL-C, TG, fasting glucose, insulin, HOMA-IR, and 50MESR at baseline and follow-up were standardized (i.e., *Z* scores were calculated: *Z* = (value – mean)/SD using sex- and age-specific means and SDs). We performed the analysis in the whole population with sex and intervention as covariates given the interaction between BMI/PBF/CRF and intervention or sex for changes in most CMR factors was not significant (Tables S1, S2). Changes in CMR factors and CRF were calculated by subtracting the results at baseline from those at follow-up. We used generalized linear regression models (GLMs) to test the difference in changes of CRF across quintiles of baseline and changes in BMI and PBF. Generalized linear regression model was used to test the difference in changes in CMR factors between sex-specific quintiles of CRF/BMI/PBF. We tested two models: (1) adjusted for classes in school as clustering effects and characteristics of individuals including age and sex as fixed effects and (2) adjusted for Model 1 plus BMI, CRF, physical activity, energy intake at baseline, puberty, birth weight, grade, household income, mother's education, father's education, mother's BMI, and father's BMI. Benjamini–Hochberg's procedure was used to control the false discovery rate at level 5% for multiple comparisons (35). Bonferroni *P*-value adjustments were performed for all pairwise comparisons.

To examine whether the association between CRF and changes in CMR factors was modified by BMI or PBF, we performed interaction analysis using GLM adjusted for covariates in Model 3. Stratified analysis was conducted for those CMR factors with a significant interaction.

We examined the mediation effect of BMI/PBF on the association between baseline and changes in CRF and changes in CMR. The direct effects of the association between CRF and changes in CMR factors and the indirect effects of this association via BMI/PBF were obtained using GLM adjusted for covariates in Model 3 of **Table 2**. The percentage of the total effect of CRF on changes in CMR factors explained by BMI/PBF was then calculated (36, 37). We used the following criteria to establish mediation: (1) the mediator was significantly associated with the exposure; (2) the exposure was significantly associated with the outcome; (3) the mediator was significantly associated with the outcome; and (4) the association between the exposure and outcome was attenuated by the mediator (21). We also examined the mediation effect of CRF on the association between BMI/PBF and CMR factors.

Sensitivity analysis was performed to test the association between CRF/BMI/PBF and changes in CMR factors in children in the control group.

Analyses were performed using SAS version 9.4 (SAS Institute Inc., Cary, NC, USA), and all *P*-values were two-sided.

## Results

### Study Population

We included 5,869 children aged 9.1 ± 1.4 (6–13) years in the final analysis. Compared with dropouts, those included in the analysis had higher blood pressure, TC, insulin, and CMRS and similar age and BMI (Table S3). Boys had higher BMI, WC, SBP, MAP, fasting glucose and HDL-C and lower PBF, TC, TG, and LDL-C than girls (Table 1, all *P* < 0.05).

**Table 1:** Baseline characteristics of boys and girls.

	**All**	**Boys**	**Girls**	*P*-value^*^
Age (years)	9.09 ± 1.40^†^	9.02 ± 1.39	9.06 ± 1.39	0.0784
BMI (kg/m^2^)	17.58 ± 3.38	16.60 ± 2.83	17.09 ± 3.16	<0.0001
WC (cm)	59.43 ± 9.73	56.07 ± 7.68	57.76 ± 8.93	<0.0001
PBF (%)	22.75 ± 5.07	25.30 ± 4.29	24.01 ± 4.87	<0.0001
SBP (mm Hg)	100.54 ± 11.09	98.15 ± 10.88	99.36 ± 11.05	<0.0001
DBP (mm Hg)	62.66 ± 8.92	63.00 ± 9.31	62.83 ± 9.11	0.15
MAP (mm Hg)	75.27 ± 8.88	74.70 ± 9.04	74.99 ± 8.96	0.0143
TC (mmol/L)	4.15 ± 0.81	4.12 ± 0.79	4.17 ± 0.84	0.0140
TG (mmol/L)	0.82 ± 0.45	0.80 ± 0.46	0.84 ± 0.44	0.0054
HDL-C (mmol/L)	1.48 ± 0.30	1.49 ± 0.31	1.46 ± 0.30	0.0005
LDL-C (mmol/L)	2.26 ± 0.62	2.23 ± 0.59	2.30 ± 0.65	<0.0001
Fasting glucose (mmol/L)	4.55 ± 0.53	4.62 ± 0.52	4.49 ± 0.54	<0.0001
CMRS (SD)	0.05 ± 2.29	0.06 ± 2.32	0.04 ± 2.26	0.76
Log HOMA-IR	−2.83 ± 0.64	−2.83 ± 0.64	−2.83 ± 0.64	0.82
Log insulin	1.66 ± 0.60	1.65 ± 0.61	1.67 ± 0.60	0.11
**CRF at baseline**				<0.0001
Unfit	1,569 (26.7)	725 (24.5)	844 (29.0)	
Fit	4,300 (73.3)	2,237 (75.5)	2,063 (71.0)	
**CRF at follow-up**				<0.0001
Unfit	1,606 (27.4)	742 (25.1)	864 (29.7)	
Fit	4,263 (72.6)	2,220 (74.9)	2,043 (70.3)	
**BMI at baseline**				<0.0001
Normal	4,626 (78.8)	2,211 (74.7)	2,415 (83.1)	
Overweight	882 (15.0)	497 (16.8)	385 (13.2)	
Obesity	361 (6.2)	254 (8.6)	107 (3.7)	
**BMI at follow-up**				<0.0001
Normal	4,119 (71.2)	1,919 (65.7)	2,200 (76.8)	
Overweight	1,285 (22.2)	730 (25.0)	555 (19.4)	
Obesity	381 (6.6)	271 (9.3)	110 (3.8)	
Physical activity (MET/week)	648.31 ± 553.23	618.53 ± 521.87	633.56 ± 538.09	0.0340
Energy (kcal/day)	1,324.74 ± 479.08	1,323.34 ± 485.33	1,324.05 ± 482.14	0.91
**Puberty**				<0.0001
Yes	168 (5.8)	10 (0.7)	158 (10.8)	
No	2,752 (94.2)	1,453 (99.3)	1,299 (89.2)	
**Grade**				0.61
1	1,151 (19.6)^‡^	574 (19.4)	577 (19.8)	
2	1,307 (22.3)	664 (22.4)	643 (22.1)	
3	1,288 (21.9)	633 (21.4)	655 (22.5)	
4	1,341 (22.8)	701 (23.7)	640 (22.0)	
5	782 (13.3)	390 (13.2)	392 (13.5)	
**Birth weight**				<0.0001
<2,500 g	147 (2.5)	60 (2.0)	87 (3.0)	
2,500–3,999 g	3,559 (60.6)	1,702 (57.5)	1,857 (63.9)	
≥4,000 g	369 (6.3)	244 (8.2)	125 (4.3)	
Missing	1,794 (30.6)	956 (32.3)	838 (28.8)	
**Mother's BMI**				0.0320
<24 kg/m^2^	3,439 (58.6)	1,702 (57.5)	1,737 (59.8)	
24–27.9 kg/m^2^	692 (11.8)	346 (11.7)	346 (11.9)	
≥28 kg/m^2^	100 (1.7)	43 (1.5)	57 (2.0)	
Missing	1,638 (27.9)	871 (29.4)	767 (26.4)	
**Father's BMI**				0.0414
<24 kg/m^2^	2,372 (40.4)	1,177 (39.7)	1,195 (41.1)	
24–27.9 kg/m^2^	1,478 (25.2)	721 (24.3)	757 (26.0)	
≥28 kg/m^2^	381 (6.5)	193 (6.5)	188 (6.5)	
Missing	1,638 (27.9)	871 (29.4)	767 (26.4)	
**Mother's education**				0.0213
<7 years	353 (6.0)	196 (6.6)	157 (5.4)	
7–12 years	2,643 (45.0)	1,270 (42.9)	1,373 (47.2)	
≥13 years	1,165 (19.9)	579 (19.5)	586 (20.2)	
Missing	1,708 (29.1)	917 (31.0)	791 (27.2)	
**Father's education**				0.10
<7 years	212 (3.6)	119 (4.0)	93 (3.2)	
7–12 years	2,667 (45.4)	1,311 (44.3)	1,356 (46.6)	
≥13 years	1,291 (22.0)	627 (21.2)	664 (22.8)	
Missing	1,699 (28.9)	905 (30.6)	794 (27.3)	
**Household income per month**				0.0219
<750 RMB	456 (7.8)	214 (7.2)	242 (8.3)	
751–1,500 RMB	1,288 (21.9)	634 (21.4)	654 (22.5)	
1,501–2,500 RMB	1,121 (19.1)	578 (19.5)	543 (18.7)	
≥2,501 RMB	1,263 (21.5)	612 (20.7)	651 (22.4)	
Missing	1,741 (29.7)	924 (31.2)	817 (28.1)	
**Intervention**				0.58
No	2,920 (49.8)	1,463 (49.4)	1,457 (50.1)	
Yes	2,949 (50.2)	1,499 (50.6)	1,450 (49.9)	

BMI, body mass index; CMR, cardiometabolic risk; CRF, cardiorespiratory fitness; DBP, diastolic blood pressure; HDL-C, high-density lipoprotein cholesterol; HOMA-IR, homeostatic model assessment of insulin resistance; LDL-C, low-density lipoprotein cholesterol; MAP, mean arterial pressure; SBP, systolic blood pressure; TC, total cholesterol; TG, triglyceride.

* t-Test was used to test the difference of continuous variables between boys and girls and χ^2^ for categorical variables.

† All such data are mean ± SD.

‡ *All such data are frequency (percentage)*.

### BMI, PBF, and CRF

The prevalence of overweight and obesity at baseline was 15.0 and 6.2%, respectively. The corresponding number at follow-up was 22.2 and 6.6%, respectively. The CRF in speed at baseline was 3.17 ± 0.42 m/s, and it increased by 0.08 ± 0.42 m/s during follow-up. The percentage of children being unfit (defined by *Z* score) was 26.7% at baseline and 27.4% at follow-up. Boys had a higher prevalence of obesity but a lower prevalence of unfitness than girls (*P* < 0.0001).

**Figure 1:**
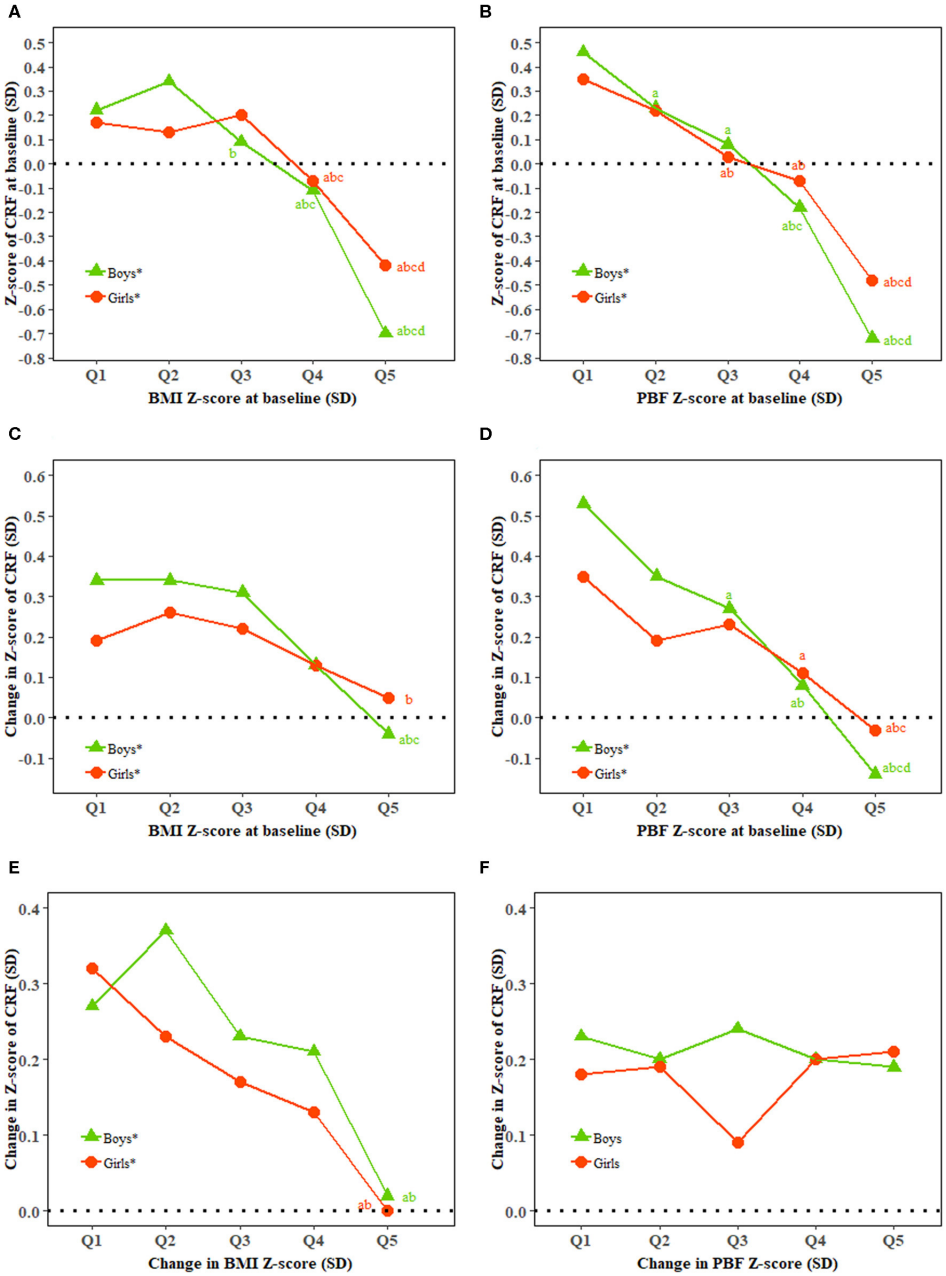
Relationship of BMI and percent body fat with cardiorespiratory fitness. BMI, body mass index; CRF, cardiorespiratory fitness; PBF, percent body fat; Q, quintile; SD, standard deviation. **(A–F)** Show baseline CRF and baseline BMI, baseline CRF and baseline PBF, change in CRF and baseline BMI, change in CRF and baseline PBF, change in CRF and change in BMI, and change in CRF and change in PBF, respectively. *Represents a significant linear trend of CRF associated with quintiles of BMI or PBF. Generalized linear regression model was used to estimate means and standard errors for each quintile of BMI and PBF adjusted for classes in school as clustering effects and characteristics of the individuals including age (additional adjustment for BMI and PBF for change in BMI and PBF and CRF at baseline for change in CRF) as fixed effects. ^abcd^Bonferroni *post hoc* test was used to examine the difference between every two quintiles of BMI and PBF with ^a^indicating significance compared with Quintile 1, ^b^indicating significance compared with Quintile 2, ^c^indicating significance compared with Quintile 3, and ^d^indicating significance compared with Quintile 4.

There was a high correlation between BMI and PBF and CRF at baseline in both boys and girls. Both BMI and PBF at baseline were inversely associated with the change in CRF in both boys and girls (all *P* < 0.0001). Change in BMI but not PBF was inversely associated with the change in CRF (Figure 1).

### CRF and Changes in CMR Factors

High baseline CRF was associated with favorable changes in all CMR factors except TC and fasting glucose after adjustment for age and sex. These CMR factors were restricted to CMRS, BMI, WC, PBF, TC, LDL-C, and HDL-C after adjustment for full confounders (all *P* < 0.025). Improved CRF was associated with favorable changes in CMRS, BMI, WC, PBF, TC, LDL-C, HDL-C, TG, and fasting glucose in the multi-variable analysis (all *P* < 0.0321; Table 2).

**Table 2:** Changes in cardiometabolic risk factors associated with baseline and change in cardiorespiratory fitness.

	**CRF at baseline (*****Z*** **score)**			**Change in CRF (*****Z*** score)^*^		
	**Participants**	**Age- and sex-adjusted β (95% CI)**	**Multi-variable-adjusted β (95% CI)^**†**^**	**Participants**	**Age- and sex-adjusted β (95% CI)**	**Multi-variable-adjusted β (95% CI)^**†**^**
Change in BMI^*^	5,777	−0.0532 (−0.0677, −0.0386)^‡^	−0.0527 (−0.0670, −0.0383)^‡^	4,846	−0.0259 (−0.0400, −0.0119)^‡^	−0.0502 (−0.0651, −0.0353)^‡^
Change in WC	5,754	−0.0528 (−0.0660, −0.0396)^‡^	−0.0456 (−0.0584, −0.0328)^‡^	4,825	−0.0018 (−0.0145, 0.0108)	−0.0237 (−0.0369, −0.0104)^‡^
Change in PBF	5,623	−0.0616 (−0.0817, −0.0416)^‡^	−0.0515 (−0.0713, −0.0318)^‡^	4,704	−0.0051 (−0.0240, 0.0138)	−0.0322 (−0.0525, −0.0119)^‡^
Change in SBP	5,755	−0.0566 (−0.0814, −0.0318)^‡^	0.0221 (−0.0033, 0.0475)	4,831	−0.0172 (−0.0425, 0.0082)	−0.0123 (−0.0392, 0.0146)
Change in DBP	5,762	−0.0473 (−0.0725, −0.0222)^‡^	0.0028 (−0.0233, 0.0289)	4,838	0.0022 (−0.0236, 0.0281)	0.0045 (−0.0234, 0.0324)
Change in MAP	5,753	−0.0535 (−0.0785, −0.0284)^‡^	0.0123 (−0.0134, 0.0380)	4,830	−0.0064 (−0.0321, 0.0193)	−0.0029 (−0.0304, 0.0246)
Change in TC	5,460	−0.0168 (−0.0357, 0.0020)	−0.0262 (−0.0461, −0.0063)^‡^	4,585	−0.0176 (−0.0364, 0.0012)	−0.0340 (−0.0543, −0.0137)^‡^
Change in HDL-C	5,451	0.1192 (0.0918, 0.1467)^‡^	0.0475 (0.0189, 0.0760)^‡^	4,579	0.0373 (0.0097, 0.0650)^‡^	0.0602 (0.0308, 0.0895)^‡^
Change in LDL-C	5,462	−0.0414 (−0.0634, −0.0194)^‡^	−0.0465 (−0.0696, −0.0235)^‡^	4,586	−0.0316 (−0.0542, −0.0091)^‡^	−0.0597 (−0.0842, −0.0353)^‡^
Change in TG	5,465	−0.0805 (−0.1030, −0.0579)^‡^	−0.0176 (−0.0408, 0.0055)	4,589	−0.0301 (−0.0522, −0.0079)^‡^	−0.0420 (−0.0655, −0.0185)^‡^
Change in fasting glucose	5,463	0.0151 (−0.0045, 0.0346)	0.0082 (−0.0125, 0.0289)	4,588	−0.0298 (−0.0498, −0.0099)^‡^	−0.0368 (−0.0585, −0.0150)^‡^
Change in insulin	4,971	−0.1025 (−0.1440, −0.0610)^‡^	−0.0011 (−0.0430, 0.0407)	4,181	0.0379 (−0.0037, 0.0794)	0.0409 (−0.0028, 0.0845)
Change in HOMA-IR	4,966	−0.0969 (−0.1368, −0.0570)^‡^	0.0012 (−0.0393, 0.0417)	4,176	0.0264 (−0.0137, 0.0665)	0.0285 (−0.0136, 0.0707)
Change in CMRS^‡^	4,948	−0.2937 (−0.3601, −0.2274)^‡^	−0.0946 (−0.1590, −0.0302)^‡^	4,179	−0.0994 (−0.1635, −0.0353)^‡^	−0.1552 (−0.2201, −0.0903)^‡^

BMI, body mass index; CMR, cardiometabolic risk; CRF, cardiorespiratory fitness; DBP, diastolic blood pressure; HDL-C, high-density lipoprotein cholesterol; HOMA-IR, homeostatic model assessment of insulin resistance; LDL-C, low-density lipoprotein cholesterol; MAP, mean arterial pressure; SBP, systolic blood pressure; TC, total cholesterol; TG, triglyceride.

* Changes in CMR factors and cardiorespiratory fitness were calculated by subtracting the results at baseline from those at follow-up.

† GLM was used to estimate multi-variable-adjusted β and 95% CIs of cardiometabolic risk factors associated with cardiorespiratory fitness. Multi-variable-adjusted analysis was adjusted for children within classes in school as clustering effects and characteristics of individuals including age, sex, corresponding CMR factor at baseline, puberty, grade, intervention, BMI, physical activity, energy intake, birth weight, household income, mother's education, father's education, mother's BMI, and father's BMI as fixed effects.

‡ *Indicates significant associations. Benjamini–Hochberg procedure was used to control the false discovery rate at level 5% for multiple comparisons with the P-value cutoff point of significance was 0.0429, 0.025, 0.0179, and 0.0321 for age- and sex-adjusted analysis (baseline CRF), multi-variable-adjusted analysis (baseline CRF), age- and sex-adjusted analysis (change in CRF), and multi-variable-adjusted analysis (change in CRF), respectively*.

### BMI and Changes in CMR Factors

Baseline BMI was positively associated with changes in CMRS, BMI, WC, PBF, SBP, DBP, MAP, TG, insulin, and HOMA-IR after adjustment for confounders (all *P* < 0.0462). Baseline BMI was inversely associated with changes in HDL-C, TC, and fasting glucose.

A low increase in BMI was associated with favorable changes in all CMR factors (all *P* < 0.05). Each SD increase in BMI was associated with a 1.2874 [95% confidence interval (CI), 1.1713, 1.4035] SD increase in CMRS after adjustment for confounders (Table 3).

**Table 3:** Changes in cardiometabolic risk factors associated with baseline and changes in BMI.

	**BMI at baseline (*****Z*** **score)**			**Change in BMI (*****Z*** score)^*^		
	**Participants**	**Age- and sex-adjusted β (95% CI)**	**Multi-variable-adjusted β (95% CI)^**†**^**	**Participants**	**Age- and sex-adjusted β (95% CI)**	**Multi-variable-adjusted β (95% CI)^**†**^**
Change in WC^*^	5,753	0.1932 (0.1652, 0.2211)^‡^	0.1821 (0.1543, 0.2099)^‡^	5,737	0.4647 (0.4445, 0.4849)^‡^	0.5824 (0.5629, 0.6019)^‡^
Change in PBF	5,623	0.2254 (0.1975, 0.2534)^‡^	0.2130 (0.1845, 0.2416)^‡^	5,621	0.8679 (0.8358, 0.8999)^‡^	0.8640 (0.8326, 0.8953)^‡^
Change in SBP	5,753	0.2614 (0.2358, 0.2870)^‡^	0.2529 (0.2253, 0.2806)^‡^	5,733	0.2209 (0.1749, 0.2669)^‡^	0.2568 (0.2118, 0.3017)^‡^
Change in DBP	5,760	0.1702 (0.1448, 0.1956)^‡^	0.1569 (0.1294, 0.1843)^‡^	5,740	0.1322 (0.0858, 0.1785)^‡^	0.1455 (0.0992, 0.1917)^‡^
Change in MAP	5,751	0.2252 (0.1996, 0.2507)^‡^	0.2126 (0.1851, 0.2402)^‡^	5,731	0.1818 (0.1354, 0.2281)^‡^	0.2058 (0.1601, 0.2515)^‡^
Change in TC	5,458	−0.0230 (−0.0421, −0.0040)^‡^	−0.0259 (−0.0466, −0.0053)^‡^	5,372	0.0653 (0.0295, 0.1011)^‡^	0.0609 (0.0249, 0.0969)^‡^
Change in HDL-C	5,449	−0.2129 (−0.2406, −0.1853)^‡^	−0.1880 (−0.2179, −0.1581)^‡^	5,363	−0.2302 (−0.2823, −0.1781)^‡^	−0.2283 (−0.2794, −0.1771)^‡^
Change in LDL-C	5,460	−0.0067 (−0.0289, 0.0156)	−0.0093 (−0.0332, 0.0146)	5,374	0.1166 (0.0750, 0.1582)^‡^	0.1164 (0.0749, 0.1579)^‡^
Change in TG	5,463	0.2049 (0.1821, 0.2277)^‡^	0.1932 (0.1684, 0.2180)^‡^	5,377	0.2640 (0.2216, 0.3065)^‡^	0.2729 (0.2311, 0.3146)^‡^
Change in fasting glucose	5,461	−0.0263 (−0.0459, −0.0067)^‡^	−0.0257 (−0.0470, −0.0043)^‡^	5,375	0.0599 (0.0228, 0.0971)^‡^	0.0542 (0.0168, 0.0915)^‡^
Change in insulin	4,969	0.4344 (0.3893, 0.4796)^‡^	0.4157 (0.3676, 0.4638)^‡^	4,890	0.2985 (0.2212, 0.3758)^‡^	0.3589 (0.2835, 0.4342)^‡^
Change in HOMA-IR	4,964	0.3999 (0.3570, 0.4429)^‡^	0.3833 (0.3373, 0.4293)^‡^	4,885	0.2940 (0.2194, 0.3686)^‡^	0.3428 (0.2700, 0.4157)^‡^
Change in CMRS^‡^	4,948	1.0600 (0.9801, 1.1400)^‡^	0.9962 (0.9124, 1.0799)^‡^	4,938	1.0994 (0.9751, 1.2236)^‡^	1.2874 (1.1713, 1.4035)^‡^

BMI, body mass index; CMR, cardiometabolic risk; CRF, cardiorespiratory fitness; DBP, diastolic blood pressure; HDL-C, high-density lipoprotein cholesterol; HOMA-IR, homeostatic model assessment of insulin resistance; LDL-C, low-density lipoprotein cholesterol; MAP, mean arterial pressure; SBP, systolic blood pressure; TC, total cholesterol; TG, triglyceride.

* Changes in CMR factors were calculated by subtracting the results at baseline from those at follow-up.

† GLM was used to estimate multi-variable-adjusted β and 95% CIs of cardiometabolic risk factors associated with BMI. Multi-variable-adjusted analysis was adjusted for children within classes in school as clustering effects and characteristics of individuals including age, sex, corresponding CMR factor at baseline, puberty, grade, intervention, BMI, physical activity, energy intake, birth weight, household income, mother's education, father's education, mother's BMI, and father's BMI as fixed effects.

‡ *Indicates significant associations. Benjamini–Hochberg procedure was used to control the false discovery rate at level 5% for multiple comparisons with the P-value cutoff point of significance was 0.0462, 0.0462, 0.05, and 0.05 for age- and sex-adjusted analysis (baseline BMI), multi-variable-adjusted analysis (baseline BMI), age- and sex-adjusted analysis (change in BMI), and multi-variable-adjusted analysis (change in BMI), respectively*.

### PBF and Changes in CMR Factors

In the multi-variable analysis, low PBF at baseline was associated with favorable changes in CMRS, BMI, WC, PBF, SBP, DBP, MAP, HDL-C, TG, insulin, and HOMA-IR (all *P* < 0.0423). Percent body fat at baseline was inversely associated with the change in fasting glucose. A low increase in PBF was associated with favorable changes in all CMR factors except fasting glucose (all *P* < 0.0423, Table 4).

**Table 4:** Changes in cardiometabolic risk factors associated with baseline and changes in percent body fat.

	**PBF** ***Z*** **score at baseline**			**Change in PBF** ***Z*** **score**		
	**Participants**	**Age- and sex-adjusted β (95% CI)**	**Multi-variable-adjusted β (95% CI)^**^†^**^**	**Participants**	**Age- and sex-adjusted β (95% CI)**	**Multi-variable-adjusted β (95% CI)^**^†^**^**
Change in BMI	5,727	−0.0104 (−0.0289, 0.0081)	−0.0211 (−0.0399, −0.0023)^‡^	5,621	0.3633 (0.3493, 0.3772)^‡^	0.3951 (0.3807, 0.4095)^‡^
Change in WC	5,701	0.0837 (0.0671, 0.1003)^‡^	0.0725 (0.0556, 0.0893)^‡^	5,582	0.1605 (0.1437, 0.1772)^‡^	0.2168 (0.1995, 0.2341)^‡^
Change in SBP	5,703	0.1708 (0.1464, 0.1952)^‡^	0.1585 (0.1322, 0.1848)^‡^	5,582	0.0641 (0.0297, 0.0984)^‡^	0.1229 (0.0878, 0.1580)^‡^
Change in DBP	5,708	0.1130 (0.0884, 0.1376)^‡^	0.0996 (0.0731, 0.1261)^‡^	5,587	0.0632 (0.0286, 0.0978)^‡^	0.1001 (0.0645, 0.1358)^‡^
Change in MAP	5,700	0.1468 (0.1223, 0.1714)^‡^	0.1331 (0.1067, 0.1595)^‡^	5,579	0.0733 (0.0388, 0.1078)^‡^	0.1224 (0.0870, 0.1578)^‡^
Change in TC	5,411	0.0018 (−0.0165, 0.0202)	−0.0003 (−0.0202, 0.0195)	5,231	0.0660 (0.0400, 0.0920)^‡^	0.0735 (0.0466, 0.1004)^‡^
Change in HDL-C	5,403	−0.2071 (−0.2335, −0.1807)^‡^	−0.1825 (−0.2110, −0.1541)^‡^	5,223	0.0006 (−0.0375, 0.0386)	−0.0654 (−0.104, −0.0268)^‡^
Change in LDL-C	5,413	−0.0090 (−0.0307, 0.0126)	−0.0184 (−0.0415, 0.0047)	5,233	0.0391 (0.0089, 0.0693)^‡^	0.0429 (0.0118, 0.0739)^‡^
Change in TG	5,416	0.1864 (0.1644, 0.2085)^‡^	0.1738 (0.1499, 0.1976)^‡^	5,236	0.0758 (0.0447, 0.1068)^‡^	0.1452 (0.1137, 0.1767)^‡^
Change in fasting glucose	5,414	−0.0380 (−0.0569, −0.0192)^‡^	−0.0398 (−0.0602, −0.0194)^‡^	5,234	0.0103 (−0.0165, 0.0372)	−0.0041 (−0.0320, 0.0239)
Change in insulin	4,933	0.2859 (0.2449, 0.3268)^‡^	0.2689 (0.2252, 0.3125)^‡^	4,765	0.1783 (0.1224, 0.2341)^‡^	0.2944 (0.2374, 0.3513)^‡^
Change in HOMA-IR	4,928	0.2640 (0.2247, 0.3032)^‡^	0.2472 (0.2053, 0.2892)^‡^	4,760	0.1690 (0.1150, 0.2229)^‡^	0.2742 (0.2191, 0.3292)^‡^
Change in CMRS^‡^	4,914	0.6526 (0.5858, 0.7195)^‡^	0.5897 (0.5196, 0.6598)^‡^	4,823	0.2610 (0.1747, 0.3474)^‡^	0.5179 (0.4316, 0.6042)^‡^

BMI, body mass index; CMR, cardiometabolic risk; DBP, diastolic blood pressure; HDL-C, high-density lipoprotein cholesterol; HOMA-IR, homeostatic model assessment of insulin resistance; LDL-C, low-density lipoprotein cholesterol; MAP, mean arterial pressure; SBP, systolic blood pressure; TC, total cholesterol; TG, triglyceride.

^*^Changes in CMR factors were calculated by subtracting the results at baseline from those at follow-up.

† GLM was used to estimate multi-variable-adjusted β and 95% CIs of cardiometabolic risk factors associated with percent body fat. Multi-variable-adjusted analysis was adjusted for children within classes in school as clustering effects and characteristics of individuals including age, sex, corresponding CMR factor at baseline, puberty, grade, intervention, BMI, physical activity, energy intake, birth weight, household income, mother's education, father's education, mother's BMI, and father's BMI as fixed effects.

‡ *Indicates significant associations. Benjamini–Hochberg procedure was used to control the false discovery rate at level 5% for multiple comparisons with the P-value cutoff point of significance was 0.0346, 0.0423, 0.0385, and 0.0423 for age- and sex-adjusted analysis (baseline PBF), multi-variable-adjusted analysis (baseline PBF), age-, and sex-adjusted analysis (change in PBF), and multi-variable-adjusted analysis (change in PBF), respectively*.

### WC and Changes in CMR Factors

A low increase in WC was associated with favorable changes in all CMR factors (all *P* < 0.05, Table S4).

### Moderation Analysis

**Figure 2:**
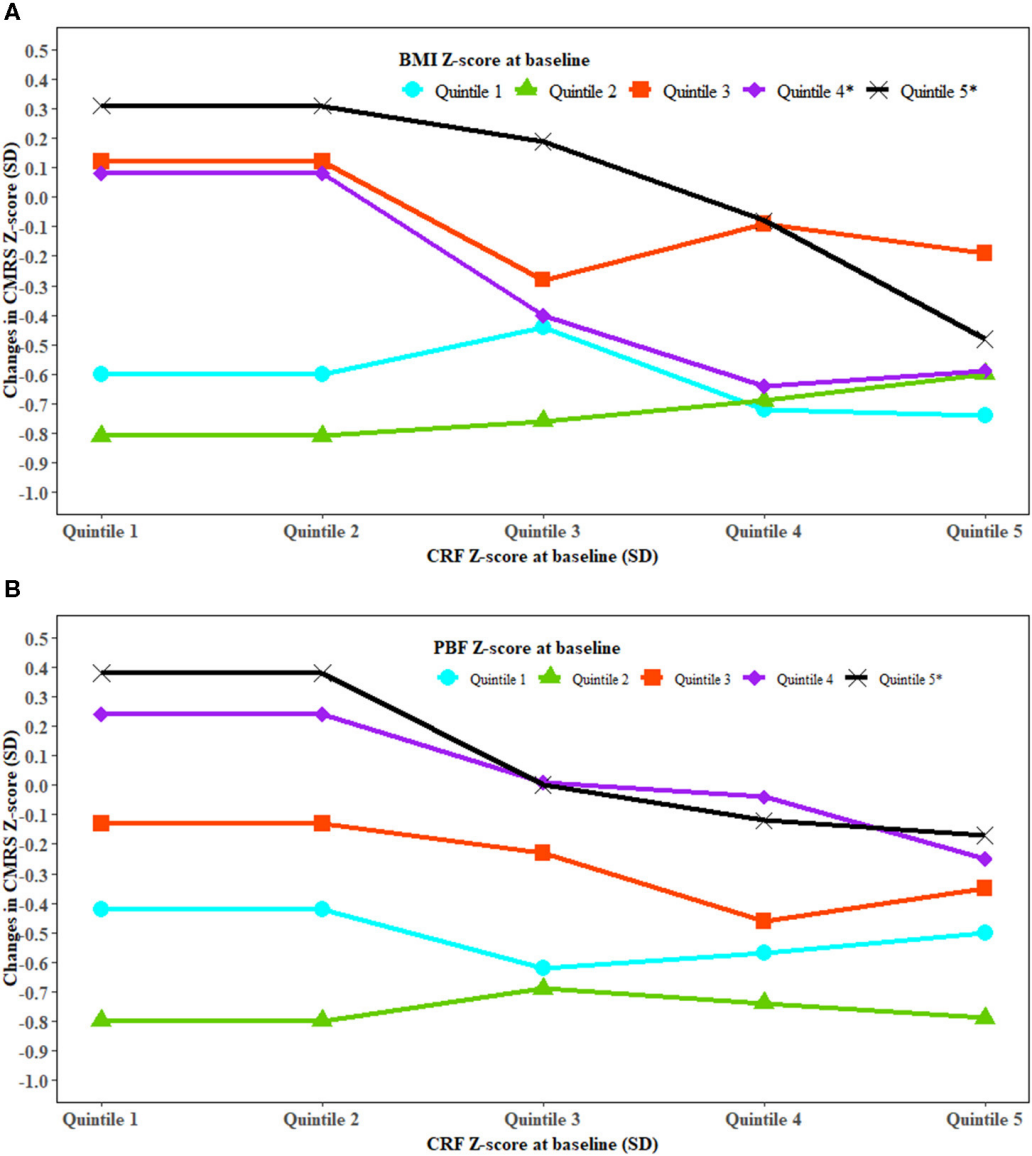
The association between cardiorespiratory fitness and change in cardiometabolic risk modified by fatness. BMI, body mass index; CRF, cardiorespiratory fitness; CMRS, cardiometabolic risk score; PBF, percent body fat; SD, standard deviation. Generalized linear regression was used to test whether the association between CRF and change in CMRS was modified by BMI **(A)** and PBF **(B)**. The analysis was adjusted for covariates in Model 3 of Table 2. *P* interaction was 0.0002 for BMI and CRF at baseline with the change in CMRS and <0.0001 for PBF and CRF at baseline with the change in CMRS. *Significant trend for changes in CMRS with CRF at baseline in the specific quintiles of BMI and PBF at baseline.

Stratified analysis showed an inverse association between baseline CRF and changes in CMRS in high but not low levels of BMI or PBF (Figure 2). An inverse association between baseline CRF and change in TG was observed in high levels of baseline BMI (Figure S2). High baseline BMI and high increase in BMI were associated with a higher increase in HOMA-IR. High baseline PBF and a greater increase in PBF were associated with a higher increase in CMRS (Figure S3). Improved CRF was associated with decreased BMI and WC in those with low but not high levels of baseline CRF (Figure S4). Improved CRF and a low increase in PBF were associated with a lower increase in fasting glucose (Figure S5).

### Mediation Analysis

The percentage of the total effect of baseline CRF on changes in CMRS, TG, HDL-C, PBF, and WC mediated by baseline BMI was 66.0, 61.6, 40.3, 20.7, and 9.2%, respectively. Changes in BMI explained 71.4, 64.1, 26.3, 22.2, 22.0, 18.8, 6.6, and 3.4% of the association between changes in CRF and changes in SBP, WC, HDL-C, CMRS, TC, TG, LDL-C, and fasting glucose, respectively. Baseline PBF explained 51.9–68.2% of the association between baseline CRF and changes in HDL-C, CMRS, and TG.

**Figure 3:**
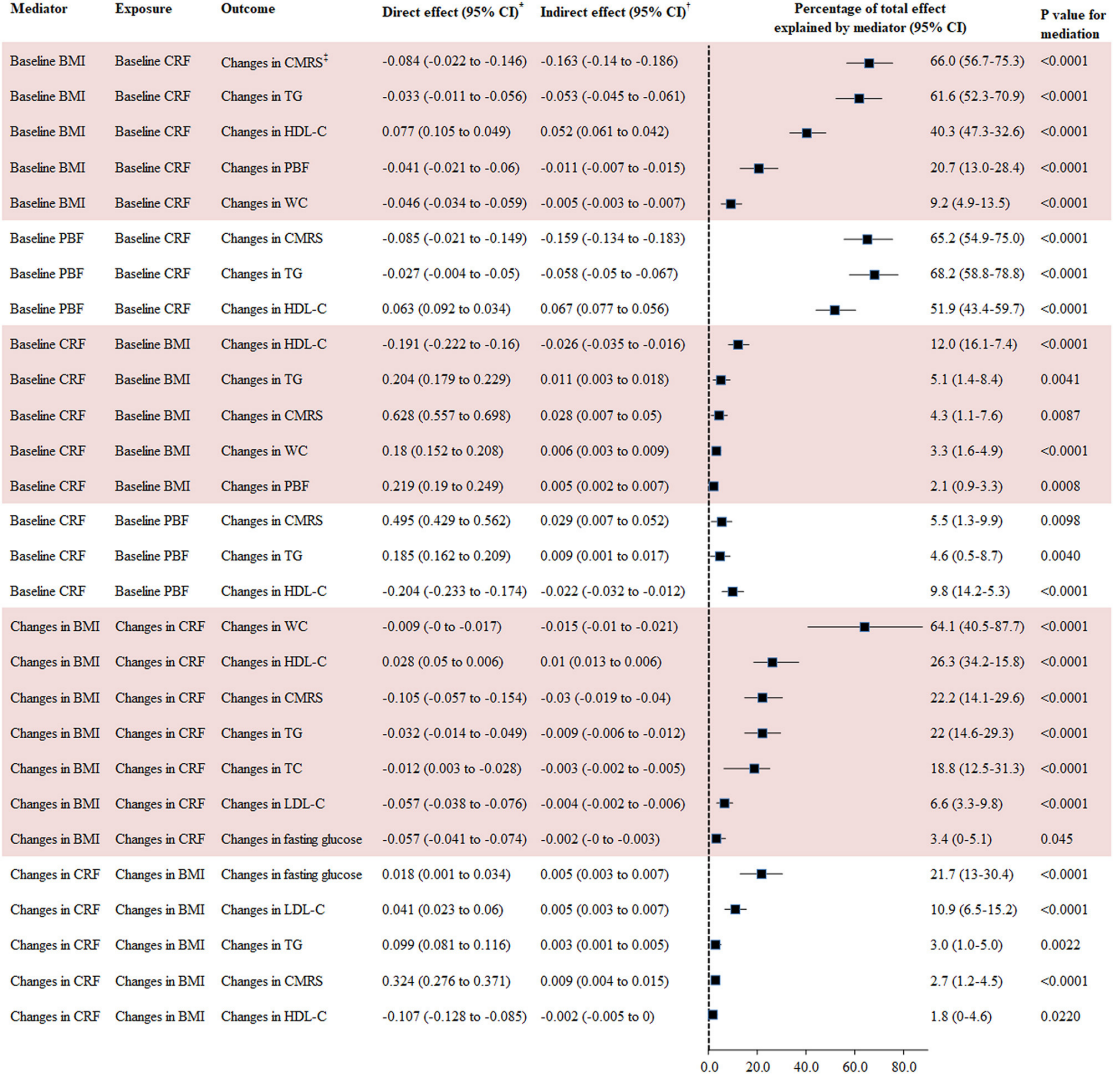
Mediation analysis for BMI, PBF, and CRF with changes in cardiometabolic risk factors. BMI, body mass index; CI, confidence interval; CMRS, cardiometabolic risk score; CRF, cardiorespiratory fitness; HDL-C, high-density lipoprotein cholesterol; LDL-C, low-density lipoprotein cholesterol; TC, total cholesterol; TG, triglyceride. *Direct effect represents the direct association between exposures and outcomes using GLM adjusted for covariates in Model 3 of Table 2. ^†^Indirect effect represents the association between exposures and outcomes via mediators using GLM adjusted for covariates in Model 3 of Table 2. CMRS was calculated by summing age- and sex-specific Z scores of the average of SBP and DBP, fasting glucose, HDL-C (multiplied by −1), and TG. Waist circumference was not taken into consideration for calculating this score in the mediation analysis for CRF and BMI with CMRS given the high correlation between WC and BMI.

Baseline CRF was a significant mediator for the association between baseline BMI and changes in CMRS (mediated by 4.3%), TG (5.1%), and HDL-C (12.0%). Changes in CRF were a significant mediator for the association between changes in BMI and changes in CMRS, TG, HDL-C, LDL-C, and fasting glucose with the mediation percentage ranging from 1.8 to 21.7% (Figure 3).

### Sensitivity Analysis

In the multi-variable analysis among children in the control group, improved CRF was associated with favorable changes in CMRS, BMI, WC, TC, HDL-C, LDL-C, TG, and fasting glucose (Table S5). A low increase in BMI was associated with favorable changes in all CMR factors except fasting glucose (Table S6). The results for associations of PBF with changes in CMR factors were like those observed in the total population (Table S7).

## Discussion

In this longitudinal analysis with large sample size, we found both fatness and CRF were independent predictors for changes in CMR factors in children. Baseline fatness was more predictive of changes in blood pressure, insulin, and HOMA-IR, and CRF was more predictive of changes in TC and LDL-C, suggesting CRF deserves scrutiny as a surrogate for fatness in predicting CMR factors. Fatness was a major mediator for the association between CRF and CMR, whereas CRF was a minor or a moderate mediator for the association between fatness and CMR. The inverse association between baseline CRF and change in CMRS and TG was more pronounced in obese children, and the inverse association between improved CRF and change in BMI and WC was more evident in unfit children.

We found boys had a lower prevalence of unfitness but a higher prevalence of obesity than girls. This is consistent with a systematic review of 1,142,026 children and youth from 50 countries showing that boys consistently had better performance in CRF than girls at each age group (32). Likely, a nationally representative survey of Chinese children reported that boys were two times more likely to be obese than girls (38). The increasing prevalence of obesity and the decreasing prevalence of healthy CRF in children worldwide as well as in China suggest it is imperative to intervene on lifestyle factors to address this problem (32, 39). In particular, resistance plus aerobic training may help result in substantial improvement in fatness and fitness in children (40, 41).

The inverse association between BMI and CRF in children has been well-established (42, 43). Likely, we found that both baseline and changes in BMI were inversely associated with changes in CRF. Some studies demonstrated that body fat mass might be more predictive of CRF compared with BMI in children (44, 45). We found the lower baseline PBF, the higher baseline CRF, and the greater increase in CRF during follow-up, but changes in PBF were not significantly associated with changes in CRF. Further study needs to examine whether changes in BMI than PBF are a better predictor for changes in CRF. Our study suggests a strong inverse association between BMI and PBF and CRF in children.

The beneficial effect of high CRF on fatness reduction in children has been well-known; however, there is less convincing evidence on other CMR factors (9). Most of the previous studies investigating the association between CRF and CMR factors were conducted in children from European countries, the United States, and Australia (9). Our study with a large sample size confirmed the beneficial effect of high baseline CRF on BMI, WC, and PBF, as well as CMRS, TC, HDL-C, LDL-C, and TG independent of corresponding CMR factor and BMI at baseline. A few longitudinal studies have investigated the association between change in CRF and changes in multiple CMR factors (9, 15, 17, 19). A 2-year longitudinal study of 1,199 European children aged 6–11 years demonstrated that low CRF was associated with higher CMRS, WC, blood lipids, and HOMA-IR independent of obesity (15). Data from another 2-year longitudinal study in 365 schoolchildren aged 7–11 years from Denmark found that change in CRF was inversely associated with the change in CMRS independent of obesity (19). Data from the HEALTHY Study of 3,514 children from the United States demonstrated that an increase in fitness was associated with favorable changes in TC, HDL-C, LDL-C, and CMRS among boys, and HDL-C among girls (17). However, these studies failed to control the corresponding CMR factor or CRF at baseline in their analysis, and no longitudinal studies from Asia have reported an association between CRF and multiple CMR factors in children. Our study showed that improved CRF was associated with favorable changes in adiposity, lipids, fasting glucose, and CMRS independent of BMI and corresponding CMR factor at baseline. One possible explanation for CRF being more predictive of changes in CMR factors in our study is the large sample size with large enough variation of baseline and change in CRF to test the significance. Furthermore, we used a test to estimate CRF different from that of previous studies. Although data from longitudinal studies in children are limited, previous studies in adults are supportive of our findings regarding the favorable effect of CRF improvement on CMR reduction (46, 47).

More studies from a larger range of countries have investigated the association between fatness and CMR factors in children compared with those studies associating CRF with CMR factors (48). There was strong evidence for the positive association between obesity and CMR factors (7, 15–17). In line with this, our study showed BMI, PBF, and WC at baseline were all positively associated with changes in CMRS, blood pressure, TG, insulin, and HOMA-IR and inversely associated with HDL-C. Although any of these obesity markers at baseline were not predictive of changes in TC or LDL-C in our study, a higher increase in each of them was associated with a higher increase in TC and LDL-C. Several longitudinal studies found baseline BMI was not significantly associated with the change in fasting glucose (7). However, we found an inverse association between baseline BMI and PBF and change in fasting glucose, even though the difference between the highest and lowest quintile was minimal (SD, 0.09). The significance of this minimal difference may be partly attributed to our large sample size. We also found that a decrease in BMI was associated with a favorable change in fasting glucose suggesting BMI reduction may be beneficial for glucose reduction. Our study agrees with previous studies showing that fatness measured in different ways was positively associated with changes in CMR factors (7).

Previous longitudinal studies from Europe and the United States have shown that BMI is a stronger predictor of changes in CMR factors than CRF (15–17). Although we found that baseline BMI/PBF/WC is predictive of changes in more CMR factors, baseline CRF is a better predictor for LDL-C and TC, suggesting CRF deserves concern as a surrogate for fatness in predicting CMR factors, whereas baseline CRF and BMI/PBF/WC are all strong predictors for TG, HDL-C, and CMRS, and changes in CRF and BMI/PBF/WC are each predictive of changes in SBP, TC, HDL-C, LDL-C, TG, fasting glucose, and CMRS, suggesting they may highly interact on CMR factors. Several cross-sectional studies from Europe have examined the extent to which the association between CRF and one or several CMR factors is explained by BMI (21, 22, 49). A cross-sectional study of 1,158 schoolchildren aged 8–11 years showed that BMI explained ~40% of the association between CRF and CMRS (21). Another cross-sectional study of 237 children and 260 adolescents demonstrated that 55.7–100% of the total effect of CRF on CMRS was mediated by BMI (22). Similarly, our mediation analysis showed that BMI and PBF were both a full or major mediator for the association between CRF and CMR factors. In contrast, CRF was a significant mediator for the association between BMI and PBF and CMR, even though the effect was relatively small. A cross-sectional analysis of 1,604 school children aged 4–7 years showed BMI acts as a full mediator in the association between CRF and MAP in boys at 62.3% and a partial mediator in girls at 35.2% (49). We found the inverse association between baseline CRF and changes in SBP was attenuated or even reversed after adjustment for BMI, suggesting the beneficial effect of high CRF may largely depend on BMI. Our mediation analysis based on longitudinal data with a large sample size suggests CRF and fatness highly interacted on the change in CMR factors. Given that these relevant studies were conducted in Europe and China (our study), more longitudinal studies from other countries are needed to examine the mediation effect of CRF on the association between fatness and CMR factors.

A few previous studies have investigated whether the association between CRF and CMR factors was modified by fatness. A pooled study of cross-sectional data from three projects with 1,247 children aged 8–11 years from Spain found an association between CRF and CMR factors was more evident in obese children (23). Similarly, a longitudinal analysis of 1,792 Australian participants aged 7–15 years reported that an increased risk of metabolic syndrome with lower CRF was only observed in the highest tertile of WC, although the interaction was not significant (*P* = 0.61) (20). Data from the HEALTHY Study of American children observed an additive effect of BMI and fitness on change in HDL-C (17). Consistently, we found the beneficial effect of high CRF on CMRS and TG reduction in the two highest quintiles of baseline BMI only. Our study also showed improved CRF was associated with a lower increase in BMI and WC in those with low baseline CRF only. This suggests encouraging children especially those with obesity or low CRF involved in exercise may significantly reduce the risk of CMR factors. However, for other CMR factors, the favorable change associated with high CRF was independent of BMI emphasizing the importance of supporting all children to take part in physical activity.

Strengths of the present study included its large sample size, multiple CMR factors examined, and various covariates adjusted for. Our study uniquely evaluated the percentage of the association between CRF and CMR explained by fatness and whether CRF was a mediator for the association between fatness and CMR in children based on longitudinal data.

The present study has several limitations. First, the relatively short time of follow-up (1 year) of our study may not be a long-enough duration to judge on the association between CRF and changes in CMR factors in children. However, we found both high baseline CRF and improvements in CRF were associated with favorable changes in CMR factors, suggesting the reliability of our findings. Second, our analysis as an observational design was based on a clinical trial, which might be biased even though the intervention was treated as a covariate in the multi-variable analysis. However, the repeated analysis in the control group showed similar results as those of the total population, suggesting that our findings are reliable. Although 50MESR has been strongly associated with CMR factors in our data and previous studies (31, 50, 51), it does not directly measure aerobic fitness.

In conclusion, the present longitudinal study demonstrates both CRF and fatness are independent predictors of changes in CMR factors in children. Although baseline fatness was predictive of changes in more CMR factors, CRF was more predictive of changes in TC and LDL-C, suggesting CRF deserves scrutiny as a surrogate for fatness in predicting CMR factors. Fatness largely mediated the association between CRF and changes in CMR factors, whereas CRF was a minor to a moderate mediator for the association between fatness and CMR factors. The favorable effect of improved CRF on changes in CMR factors was more likely to be manifested in obese or unfit children, emphasizing the importance of taking part in exercises in these subgroups.

## Data Availability Statement

The datasets generated for this study are available on request to the corresponding author.

## Ethics Statement

The studies involving human participants were reviewed and approved by Ethical Review Committee of the National Institute for Nutrition and Food Safety, Chinese Center for Disease Control and Prevention. Written informed consent to participate in this study was provided by the participants' legal guardian/next of kin.

## Author Contributions

GM designed the research. GM, YiL, JM, GX, HG, TL, and WL conducted research. XS and YaL analyzed data. XS wrote the initial draft of the manuscript. XS, YaL, HX, and GM revised the manuscript. All authors read and approved the final manuscript.

## Conflict of Interest

The authors declare that the research was conducted in the absence of any commercial or financial relationships that could be construed as a potential conflict of interest.
