# Supplementary material for: Independent and Interactive Associations of Fitness and Fatness With Changes in Cardiometabolic Risk in Children: A Longitudinal Analysis
Source: Front Endocrinol (Lausanne). 2020 Jun 12;11:342. doi: 10.3389/fendo.2020.00342 (PMC7304437; doi:10.3389/fendo.2020.00342)
Supplement: Supplementary file 1 [file Data_Sheet_1.docx]

**SUPPLEMENTAL MATERIAL**

**Figure S1. Flowchart for population selection**

**Figure S2. The association between baseline CRF and changes in cardiometabolic risk factors modified by baseline BMI**

**Figure S3. The association between change in BMI/PBF and changes in cardiometabolic risk factors modified by baseline BMI/PBF**

**Figure S4. The association between changes in CRF and changes in systolic and diastolic blood pressure modified by baseline CRF**

**Figure S5. The association between change in CRF and changes in fasting glucose modified by the change in PBF**

**Table S1. P values for interaction between intervention and BMI, percent body fat, and cardiorespiratory fitness for changes in cardiometabolic risk factors**

**Table S2. P values for interaction between sex and BMI, percent body fat, and cardiorespiratory for changes in cardiometabolic risk factors**

**Table S3. Differences between children who included and those who were lost to follow-up in the analysis**

**Table S4. Changes in cardiometabolic risk factors associated with baseline and changes in waist circumference**

**Table S5. Changes in cardiometabolic risk factors associated with baseline and changes in cardiorespiratory fitness in the control group**

**Table S6. Changes in cardiometabolic risk factors associated with baseline and changes in BMI in the control group**

**Table S7. Changes in cardiometabolic risk factors associated with baseline and changes in PBF in the control group**

**Figure S1. Flowchart for population selection**


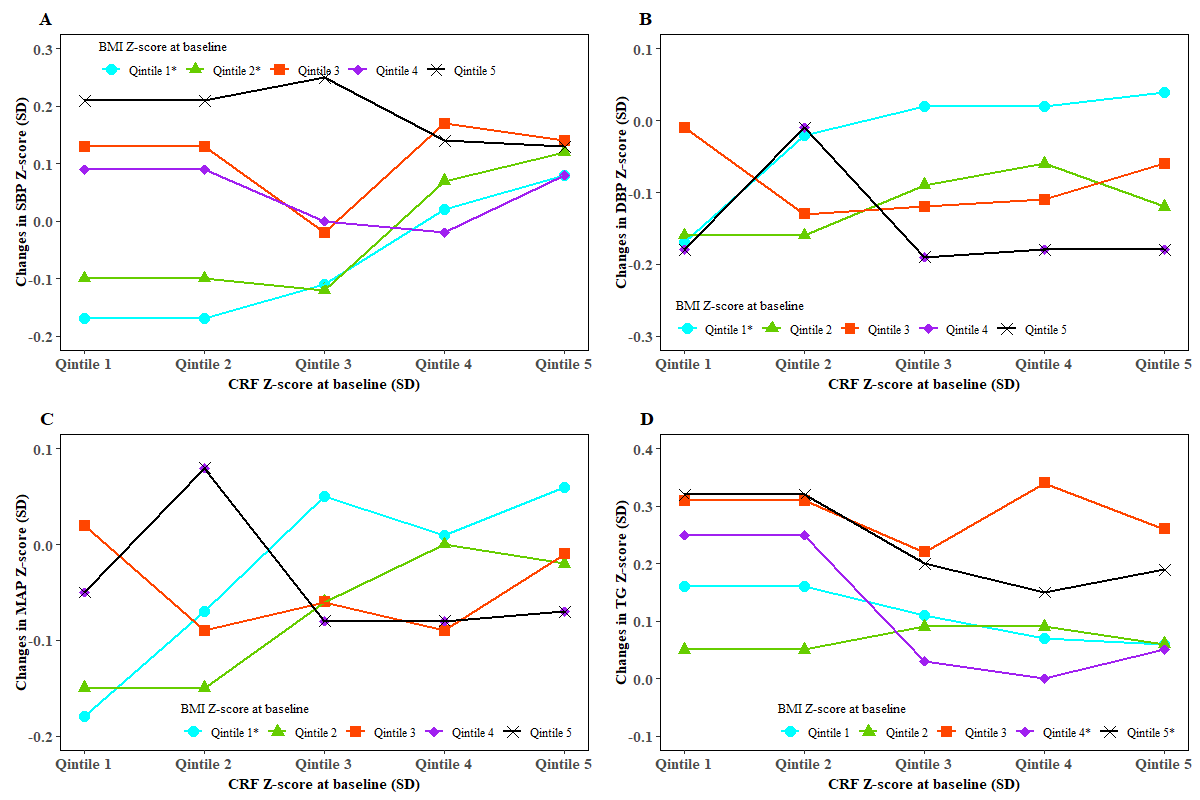


**Figure S2. The association between baseline CRF and changes in cardiometabolic risk factors modified by baseline BMI**

GLM was used to test whether the association between baseline CRF and changes in CMR factors was modified by baseline BMI. Children within classes in school as clustering effects and characteristics of the individuals including age, sex, grade, intervention, corresponding cardiometabolic risk factor, physical activity, total energy intake at baseline, birthweight, household income, mother’s education, father’s education, mother’s BMI, and father’s BMI as fixed effects were adjusted for. We did the analysis for all the CMR factors and only present those with significant moderation in this figure.

*indicates a significant trend for changes in CMR factors with baseline CRF in the specific quintiles of BMI at baseline.


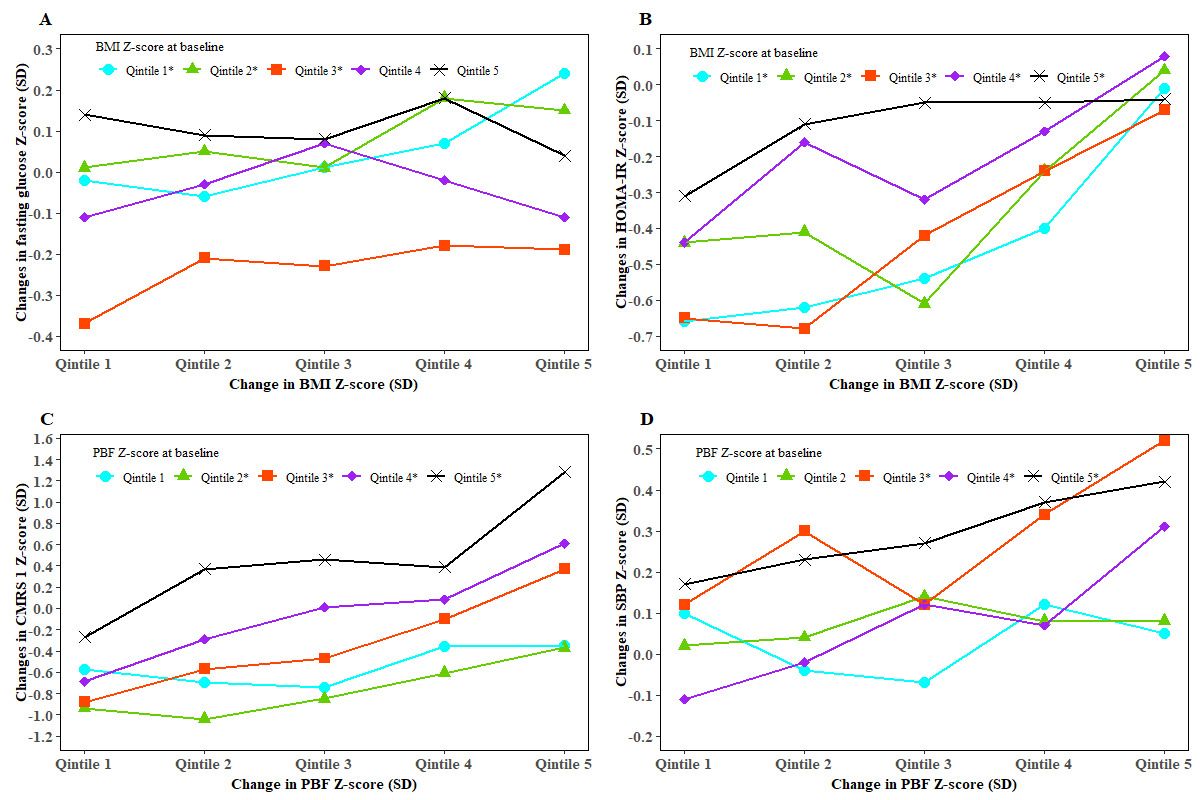


**Figure S3. The association between change in BMI/PBF and changes in cardiometabolic risk factors modified by baseline BMI/PBF**

GLM was used to test whether the association between change in BMI/PBF and changes in CMR factors was modified by baseline BMI/PBF. Children within classes in school as clustering effects and characteristics of the individuals including age, sex, grade, intervention, corresponding cardiometabolic risk factor, physical activity, total energy intake at baseline, birthweight, household income, mother’s education, father’s education, mother’s BMI, and father’s BMI as fixed effects were adjusted for. We did the analysis for all the CMR factors and only present those with significant moderation in this figure.

*indicates a significant trend for changes in CMR factors with changes in BMI/PBF in the specific quintiles of BMI/PBF at baseline.


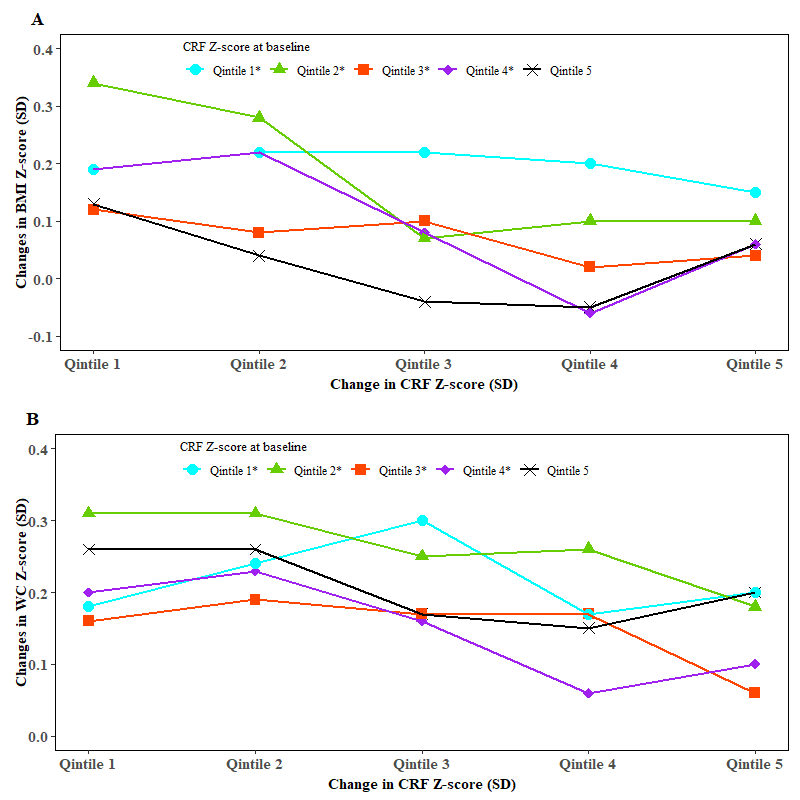


**Figure S4. The association between changes in CRF and changes in systolic and diastolic blood pressure modified by baseline CRF**

GLM was used to test whether the association between changes in CRF and changes in BMI and WC was modified by baseline CRF. Children within classes in school as clustering effects and characteristics of the individuals including age, sex, grade, intervention, corresponding cardiometabolic risk factor, physical activity, total energy intake at baseline, birthweight, household income, mother’s education, father’s education, mother’s BMI, and father’s BMI as fixed effects were adjusted for. We did the analysis for all the CMR factors and only present those with significant moderation in this figure.

*indicates a significant trend for changes in BMI and WC with changes in CRF in the specific quintiles of CRF at baseline.


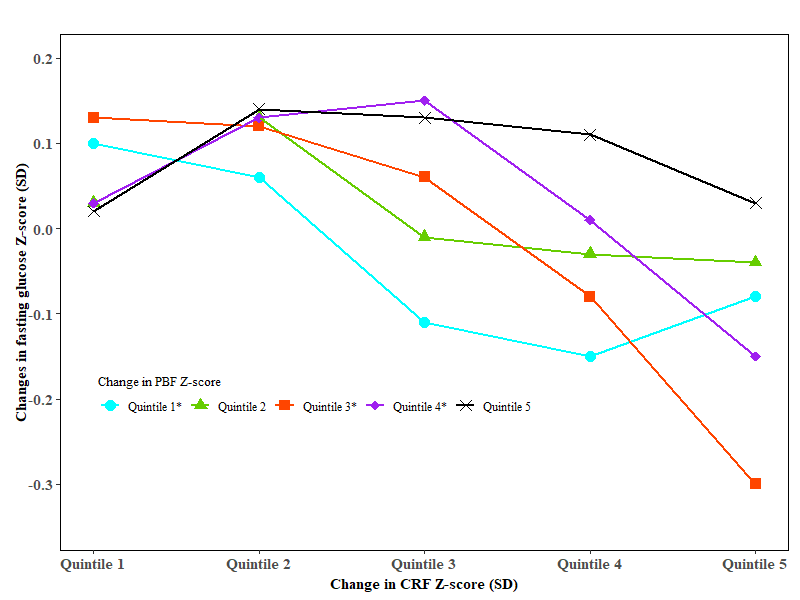


**Figure S5. The association between change in CRF and changes in fasting glucose modified by the change in PBF**

GLM was used to test whether the association between change in CRF and change in fasting glucose was modified by the change in PBF. Children within classes in school as clustering effects and characteristics of the individuals including age, sex, grade, intervention, corresponding cardiometabolic risk factor, physical activity, total energy intake at baseline, birthweight, household income, mother’s education, father’s education, mother’s BMI, and father’s BMI as fixed effects were adjusted for. We did the analysis for all the CMR factors and only present those with significant moderation in this figure.

*indicates a significant trend for changes in fasting glucose with the change in CRF in the specific quintiles of change in PBF.

**Table S1. P values for interaction between intervention and BMI, percent body fat, and cardiorespiratory fitness for changes in cardiometabolic risk factors***

| Changes in CMR factors | P interaction for intervention and BMI^†^ | P interaction for intervention and PBF | P interaction for intervention and CRF |
| --- | --- | --- | --- |
| BMI |  | 0.0474 | 0.6829 |
| WC | 0.2794 | 0.3259 | 0.5370 |
| PBF | 0.3220 |  | 0.1900 |
| SBP | **0.0061** | 0.1799 | 0.5781 |
| DBP | **0.0003** | 0.0946 | 0.1956 |
| MAP | **0.0002** | 0.0890 | 0.2397 |
| TC | 0.6183 | 0.3409 | 0.2496 |
| HDL-C | 0.6432 | 0.1334 | **0.0021** |
| LDL-C | 0.3356 | **0.0025** | **0.0003** |
| Log TG | 0.4038 | 0.6392 | 0.3830 |
| Fasting glucose | 0.0575 | 0.9454 | 0.7188 |
| Insulin | **0.0076** | 0.8382 | 0.0340 |
| HOMA-IR | **0.0100** | 0.8772 | 0.0300 |
| CMRS 1 | **0.0080** | 0.6986 | 0.2346 |
| CMRS 2 | 0.1257 | 0.9118 | 0.0773 |

BMI, body mass index; CMR, cardiometabolic risk; CRF, cardiorespiratory fitness; DBP, diastolic blood pressure; HDL-C, high-density lipoprotein cholesterol; HOMA-IR, homeostatic model assessment of insulin resistance; LDL-C, low-density lipoprotein cholesterol; MAP, mean arterial pressure; SBP, systolic blood pressure; TC, total cholesterol; TG, triglyceride.

*GLM was used to test the interaction between intervention and percent body fat, and cardiorespiratory fitness for changes in cardiometabolic risk factors adjusted for children within classes in school as clustering effects and characteristics of the individuals including age, sex, grade, corresponding CMR factor at baseline, physical activity, total energy intake, birthweight, household income, mother’s education, father’s education, mother’s BMI, and father’s BMI as fixed effects.

^†^We used Benjamin-Hochberg procedure was used to control the false discovery rate at level 5% for multiple comparisons with the P-value cut-off point of significance was 0.02, 0.003, and 0.01 for intervention and BMI, intervention and PBF, and intervention and CRF, respectively.

**Table S2. P values for interaction between sex and BMI, percent body fat, and cardiorespiratory for changes in cardiometabolic risk factors**

| Changes in CMR factors | P interaction for intervention and BMI | P interaction for intervention and PBF | P interaction for intervention and CRF |
| --- | --- | --- | --- |
| BMI |  | **<0.0001** | 0.0532 |
| WC | **<0.0001** | **<0.0001** | 0.1064 |
| PBF | **0.0012** |  | **0.0007** |
| SBP | 0.2612 | 0.4368 | 0.7829 |
| DBP | 0.5356 | 0.2277 | 0.3979 |
| MAP | 0.4571 | 0.4506 | 0.4827 |
| TC | 0.6058 | 0.8651 | 0.5287 |
| HDL-C | 0.2705 | 0.3343 | 0.6555 |
| LDL-C | 0.9251 | 0.4913 | 0.9333 |
| TG | 0.3041 | 0.4150 | 0.2886 |
| Fasting glucose | 0.1483 | 0.1710 | **0.0051** |
| Insulin | 0.4798 | 0.4306 | 0.5499 |
| HOMA-IR | 0.6292 | 0.4297 | 0.3718 |
| CMRS 1 | 0.2572 | 0.1112 | 0.3822 |
| CMRS 2 | 0.6342 | 0.8668 | 0.4287 |

BMI, body mass index; CMR, cardiometabolic risk; CRF, cardiorespiratory fitness; DBP, diastolic blood pressure; HDL-C, high-density lipoprotein cholesterol; HOMA-IR, homeostatic model assessment of insulin resistance; LDL-C, low-density lipoprotein cholesterol; MAP, mean arterial pressure; SBP, systolic blood pressure; TC, total cholesterol; TG, triglyceride.

*GLM was used to test the interaction between intervention and percent body fat, and cardiorespiratory fitness for changes in cardiometabolic risk factors adjusted for children within classes in school as clustering effects and characteristics of the individuals including age, sex, grade, corresponding CMR factor at baseline, physical activity, total energy intake, birthweight, household income, mother’s education, father’s education, mother’s BMI, and father’s BMI as fixed effects.

^†^We used Benjamin-Hochberg procedure was used to control the false discovery rate at level 5% for multiple comparisons with the P-value cut-off point of significance was 0.01, 0.0067, and 0.0067 for sex and BMI, sex and PBF, and sex and CRF, respectively.

**Table S3. Differences between children who included and those who were lost to follow-up in the analysis**

|  | All | Included in  the analysis | Lost to  follow-up | P-value* |
| --- | --- | --- | --- | --- |
| Age (years) | 9.06±1.40^†^ | 9.06±1.39 | 9.06±1.43 | 0.99 |
| BMI (kg/m^2^) | 17.08±3.21 | 17.09±3.16 | 17.02±3.37 | 0.42 |
| WC (cm) | 57.74±8.88 | 57.76±8.93 | 57.68±8.73 | 0.75 |
| % body fat mass | 23.94±4.92 | 24.01±4.87 | 23.73±5.07 | 0.0355 |
| SBP (mm Hg) | 98.86±11.15 | 99.36±11.05 | 97.28±11.33 | <0.0001 |
| DBP (mm Hg) | 62.31±9.22 | 62.83±9.11 | 60.68±9.35 | <0.0001 |
| MAP (mm Hg) | 74.48±9.08 | 74.99±8.96 | 72.86±9.25 | <0.0001 |
| Total cholesterol (mmol/L) | 4.11±0.80 | 4.15±0.81 | 4.00±0.76 | <0.0001 |
| HDL (mmol/L) | 1.49±0.31 | 1.48±0.30 | 1.55±0.33 | <0.0001 |
| LDL (mmol/L) | 2.23±0.62 | 2.26±0.62 | 2.13±0.62 | <0.0001 |
| TG (mmol/L) | 0.82±0.44 | 0.82±0.45 | 0.80±0.41 | 0.0502 |
| Fasting glucose (mmol/L) | 4.58±0.54 | 4.55±0.53 | 4.67±0.55 | <0.0001 |
| Log insulin | 1.64±0.61 | 1.66±0.60 | 1.56±0.62 | <0.0001 |
| Log HOMA-IR | 0.08±1.00 | 0.10±0.99 | -0.02±1.02 | <0.0001 |
| CMRS 1 | 0.01±2.78 | 0.08±2.77 | -0.23±2.79 | <0.0001 |
| CMRS 2 | -0.04±2.10 | 0.08±2.09 | -0.44±2.10 | <0.0001 |
| Physical activity (MET/week) | 1346.35±584.90 | 1356.28±595.23 | 1309.00±542.96 | 0.0220 |
| Energy (kcal/day) | 655.25±649.86 | 630.01±631.19 | 738.65±701.85 | <0.0001 |
| 50 M*8 shuttle run (second) | 128.65±18.64 | 128.55±18.12 | 129.61±23.23 | 0.19 |
| 51 M*8 shuttle run (Z-score) | 0.02±1.07 | 0.02±1.04 | 0.06±1.35 | 0.33 |
| Grade |  |  |  | 0.28 |
| One | 1536(19.9)^‡^ | 1151(19.6) | 385(20.8) |  |
| Two | 1713(22.2) | 1307(22.3) | 406(22.0) |  |
| Three | 1691(21.9) | 1288(21.9) | 403(21.8) |  |
| Four | 1767(22.9) | 1341(22.8) | 426(23.1) |  |
| Five | 1010(13.1) | 782(13.3) | 228(12.3) |  |
| Birth weight |  |  |  | 0.25 |
| <2500 g | 206(2.7) | 147(2.5) | 59(3.2) |  |
| 2500-3999 g | 4536(58.8) | 3559(60.6) | 977(52.9) |  |
| ≥4000 g | 474(6.1) | 369(6.3) | 105(5.7) |  |
| Missing | 2501(32.4) | 1794(30.6) | 707(38.3) |  |
| Mother's BMI |  |  |  | 0.87 |
| <24 kg/m^2^ | 4398(57.0) | 3439(58.6) | 959(51.9) |  |
| 24-27.9 kg/m^2^ | 893(11.6) | 692(11.8) | 201(10.9) |  |
| ≥28 kg/m^2^ | 124(1.6) | 100(1.7) | 24(1.3) |  |
| Missing | 2302(29.8) | 1638(27.9) | 664(35.9) |  |
| Father's BMI |  |  |  | 0.32 |
| <24 kg/m^2^ | 3048(39.5) | 2372(40.4) | 676(36.6) |  |
| 24-27.9 kg/m^2^ | 1899(24.6) | 1478(25.2) | 421(22.8) |  |
| ≥28 kg/m^2^ | 468(6.1) | 381(6.5) | 87(4.7) |  |
| Missing | 2302(29.8) | 1638(27.9) | 664(35.9) |  |
| Mother's education |  |  |  | 0.0226 |
| <7 years | 488(6.3) | 353(6.0) | 135(7.3) |  |
| 7-12 years | 3366(43.6) | 2643(45.0) | 723(39.1) |  |
| ≥13 years | 1473(19.1) | 1165(19.9) | 308(16.7) |  |
| Missing | 2390(31.0) | 1708(29.1) | 682(36.9) |  |
| Father's education |  |  |  | 0.91 |
| <7 years | 297(3.8) | 212(3.6) | 85(4.6) | 0.91 |
| 7-12 years | 3369(43.7) | 2667(45.4) | 702(38.0) | 0.91 |
| ≥13 years | 1668(21.6) | 1291(22.0) | 377(20.4) | 0.91 |
| Missing | 2383(30.9) | 1699(28.9) | 684(37.0) |  |
| Household income per month |  |  |  | 0.0009 |
| <750 RMB | 611(7.9) | 456(7.8) | 155(8.4) |  |
| 751-1500 RMB | 1661(21.5) | 1288(21.9) | 373(20.2) |  |
| 1501-2500 RMB | 1447(18.8) | 1121(19.1) | 326(17.6) |  |
| ≥2501 RMB | 1556(20.2) | 1263(21.5) | 293(15.9) |  |
| Missing | 2442(31.6) | 1741(29.7) | 701(37.9) |  |
| Intervention |  |  |  | 0.0071 |
| No | 3773(48.9) | 2920(49.8) | 853(46.2) |  |
| Yes | 3944(51.1) | 2949(50.2) | 995(53.8) |  |

BMI, body mass index; CMR, cardiometabolic risk; CRF, cardiorespiratory fitness; DBP, diastolic blood pressure; HDL-C, high-density lipoprotein cholesterol; HOMA-IR, homeostatic model assessment of insulin resistance; LDL-C, low-density lipoprotein cholesterol; MAP, mean arterial pressure; SBP, systolic blood pressure; TC, total cholesterol; TG, triglyceride.

*T-test was used to test the difference of continuous variables between boys and girls and Chi-square for categorical variables.

^†^All such data were mean ± standard deviation.

^‡^All such data were frequency (percentage).

**Table S4.** **Changes in cardiometabolic risk factors associated with baseline and changes in waist circumference**

|  | WC Z-score at baseline | | |  | Change in WC Z-score | | |
| --- | --- | --- | --- | --- | --- | --- | --- |
|  | Participants | Age- and sex-adjusted β | Multivariable-adjusted β^†^ |  | Participants | Age- and sex-adjusted β | Multivariable-adjusted β^†^ |
| Change in BMI | 5758 | 0.4265(0.3976,0.4554)^‡^ | 0.4211(0.3923,0.4499)^‡^ |  | 5737 | 0.5584(0.5337,0.5830)^‡^ | 0.6441(0.6225,0.6656)^‡^ |
| Change in PBF | 5604 | 0.2689(0.2445,0.2933)^‡^ | 0.2612(0.2362,0.2862)^‡^ |  | 5582 | 0.3902(0.3529,0.4274)^‡^ | 0.4538(0.4180,0.4897)^‡^ |
| Change in SBP | 5735 | 0.2838(0.2584,0.3092)^‡^ | 0.2810(0.2535,0.3084)^‡^ |  | 5710 | 0.2017(0.1509,0.2525)^‡^ | 0.2366(0.1872,0.2860)^‡^ |
| Change in DBP | 5742 | 0.1883(0.1632,0.2135)^‡^ | 0.1784(0.1512,0.2056)^‡^ |  | 5717 | 0.1280(0.0767,0.1793)^‡^ | 0.1418(0.0907,0.1930)^‡^ |
| Change in MAP | 5733 | 0.2470(0.2217,0.2723)^‡^ | 0.2389(0.2116,0.2663)^‡^ |  | 5708 | 0.1670(0.1159,0.2182)^‡^ | 0.1920(0.1417,0.2422)^‡^ |
| Change in TC | 5440 | -0.0219(-0.0406,-0.0033)^‡^ | -0.0252(-0.0454,-0.0049)^‡^ |  | 5348 | 0.0801(0.0412,0.1190)^‡^ | 0.0698(0.0305,0.1090)^‡^ |
| Change in HDL-C | 5432 | -0.2330(-0.2601,-0.2059)^‡^ | -0.2124(-0.2417,-0.1831)^‡^ |  | 5340 | -0.2327(-0.2895,-0.1759)^‡^ | -0.2325(-0.2881,-0.1768)^‡^ |
| Change in LDL-C | 5442 | -0.0030(-0.0248,0.0188) | -0.0058(-0.0293,0.0177) |  | 5350 | 0.1748(0.1295,0.2201)^‡^ | 0.1661(0.1208,0.2113)^‡^ |
| Change in TG | 5445 | 0.2194(0.1971,0.2418)^‡^ | 0.2102(0.1859,0.2345)^‡^ |  | 5353 | 0.3117(0.2653,0.3581)^‡^ | 0.3172(0.2716,0.3628)^‡^ |
| Change in fasting glucose | 5443 | -0.0158(-0.0351,0.0034) | -0.0127(-0.0336,0.0083) |  | 5351 | 0.0792(0.0388,0.1196)^‡^ | 0.0736(0.0328,0.1143)^‡^ |
| Change in insulin | 4952 | 0.4347(0.3899,0.4795)^‡^ | 0.4160(0.3683,0.4637)^‡^ |  | 4869 | 0.2751(0.1922,0.3580)^‡^ | 0.3332(0.2523,0.4140)^‡^ |
| Change in HOMA-IR | 4947 | 0.4031(0.3605,0.4457)^‡^ | 0.3871(0.3415,0.4326)^‡^ |  | 4864 | 0.2764(0.1964,0.3563)^‡^ | 0.3251(0.2469,0.4032)^‡^ |
| Change in CMRS^‡^ | 4948 | 1.2593(1.1780,1.3406)^‡^ | 1.2090(1.1238,1.2942)^‡^ |  | 4948 | 1.5771(1.4443,1.7099)^‡^ | 1.6491(1.5281,1.7701)^‡^ |

BMI, body mass index; CMR, cardiometabolic risk; CRF, cardiorespiratory fitness; DBP, diastolic blood pressure; HDL-C, high-density lipoprotein cholesterol; HOMA-IR, homeostatic model assessment of insulin resistance; LDL-C, low-density lipoprotein cholesterol; MAP, mean arterial pressure; SBP, systolic blood pressure; TC, total cholesterol; TG, triglyceride.

*Changes in CMR factors and cardiorespiratory fitness were calculated by subtracting the results at baseline from those at follow-up.

^†^GLM was used to estimate multivariable-adjusted β and 95% CIs of cardiometabolic risk factors associated with waist circumference. Multivariable-adjusted analysis was adjusted for children within classes in school as clustering effects and characteristics of individuals including age, sex, corresponding CMR factor at baseline, puberty, grade, intervention, BMI, physical activity, energy intake, birthweight, household income, mother’s education, father’s education, mother’s BMI, and father’s BMI as fixed effects.

^‡^ Indicates significant associations. Benjamin-Hochberg procedure was used to control the false discovery rate at level 5% for multiple comparisons with the P-value cut-off point of significance was 0.0385, 0.0385, 0.05, and 0.05 for age- and sex-adjusted analysis (baseline WC), multivariable-adjusted analysis (baseline WC), age- and sex-adjusted analysis (change in WC), and multivariable-adjusted analysis (change in WC), respectively.

**Table S5. Changes in cardiometabolic risk factors associated with baseline and changes in cardiorespiratory fitness in the control group**

|  | Baseline CRF (Z-score) | | |  | Change in CRF (Z-score)* | | |
| --- | --- | --- | --- | --- | --- | --- | --- |
|  | Participants | Age- and sex-adjusted | Multivariable-adjusted^†^ |  | Participants | Age- and sex-adjusted | Multivariable-adjusted^†^ |
| Change in BMI | 2880 | -0.0640 (-0.0864,-0.0415)^‡^ | -0.0605 (-0.0828,-0.0383)^‡^ |  | 2334 | -0.0358 (-0.0554,-0.0161)^‡^ | -0.0515 (-0.0712,-0.0317)^‡^ |
| Change in WC | 2870 | -0.0662 (-0.0865,-0.0458)^‡^ | -0.0543 (-0.0740,-0.0345)^‡^ |  | 2326 | -0.0057 (-0.0235,0.0120) | -0.0211 (-0.0385,-0.0037)^‡^ |
| Change in PBF | 2805 | -0.0483 (-0.0772,-0.0194)^‡^ | -0.0351 (-0.0636,-0.0066)^‡^ |  | 2265 | -0.0052 (-0.0299,0.0196) | -0.0223 (-0.0478,0.0033) |
| Change in SBP | 2878 | -0.0623 (-0.1016,-0.0231)^‡^ | 0.0325 (-0.0081,0.0731) |  | 2334 | -0.0161 (-0.0531,0.0210) | -0.0056 (-0.0440,0.0328) |
| Change in DBP | 2878 | -0.0425 (-0.0816,-0.0033)^‡^ | 0.0200 (-0.0207,0.0608) |  | 2334 | -0.0026 (-0.0399,0.0347) | 0.0027 (-0.0362,0.0415) |
| Change in MAP | 2876 | -0.0507 (-0.0902,-0.0112)^‡^ | 0.0292 (-0.0117,0.0700) |  | 2332 | -0.0074 (-0.0451,0.0302) | -0.0006 (-0.0396,0.0383) |
| Change in TC | 2728 | -0.0218 (-0.0500,0.0064) | -0.0328 (-0.0631,-0.0026) |  | 2199 | -0.0194 (-0.0446,0.0059) | -0.0352 (-0.0620,-0.0085)^‡^ |
| Change in HDL-C | 2726 | 0.0785 (0.0388,0.1182)^‡^ | -0.0013 (-0.0429,0.0404) |  | 2198 | 0.0592 (0.0233,0.0952)^‡^ | 0.0597 (0.0222,0.0971)^‡^ |
| Change in LDL-C | 2730 | -0.0903 (-0.1214,-0.0593)^‡^ | -0.0971 (-0.1300,-0.0642)^‡^ |  | 2200 | -0.0508 (-0.0809,-0.0207)^‡^ | -0.0849 (-0.1162,-0.0535)^‡^ |
| Change in log TG | 2733 | -0.0730 (-0.1071,-0.0389)^‡^ | 0.0060 (-0.0295,0.0415) |  | 2203 | -0.0480 (-0.0775,-0.0184)^‡^ | -0.0497 (-0.0803,-0.0191)^‡^ |
| Change in fasting glucose | 2731 | 0.0219 (-0.0076,0.0514) | 0.0105 (-0.0211,0.0422) |  | 2201 | -0.0431 (-0.0708,-0.0154)^‡^ | -0.0487 (-0.0781,-0.0193)^‡^ |
| Change in log insulin | 2466 | -0.0303 (-0.0922,0.0317) | 0.0657 (0.0023,0.1291) |  | 1984 | 0.0212 (-0.0374,0.0799) | 0.0412 (-0.0194,0.1018) |
| Change in HOMA-IR | 2464 | -0.0289 (-0.0882,0.0305) | 0.0640 (0.0030,0.1251) |  | 1982 | 0.0101 (-0.0458,0.0661) | 0.0286 (-0.0293,0.0866) |
| Change in CMRS | 2475 | -0.2619 (-0.3631,-0.1607)^‡^ | -0.0405 (-0.1397,0.0586) |  | 2009 | -0.1531 (-0.2385,-0.0677)^‡^ | -0.1729 (-0.2584,-0.0874)^‡^ |

BMI, body mass index; CMR, cardiometabolic risk; CRF, cardiorespiratory fitness; DBP, diastolic blood pressure; HDL-C, high-density lipoprotein cholesterol; HOMA-IR, homeostatic model assessment of insulin resistance; LDL-C, low-density lipoprotein cholesterol; MAP, mean arterial pressure; SBP, systolic blood pressure; TC, total cholesterol; TG, triglyceride.

*Changes in CMR factors and cardiorespiratory fitness were calculated by subtracting the results at baseline from those at follow-up.

^†^GLM was used to estimate multivariable-adjusted β and 95% CIs of cardiometabolic risk factors associated with cardiorespiratory fitness. Multivariable-adjusted analysis was adjusted for children within classes in school as clustering effects and characteristics of individuals including age, sex, corresponding CMR factor at baseline, puberty, grade, intervention, BMI, physical activity, energy intake, birthweight, household income, mother’s education, father’s education, mother’s BMI, and father’s BMI as fixed effects.

^‡^ Indicates significant associations. Benjamin-Hochberg procedure was used to control the false discovery rate at level 5% for multiple comparisons with the P-value cut-off point of significance was 0.0357, 0.0179, 0.0214, and 0.0286 for age- and sex-adjusted analysis (baseline CRF), multivariable-adjusted analysis (baseline CRF), age- and sex-adjusted analysis (change in CRF), and multivariable-adjusted analysis (change in CRF), respectively.

**Table S6. Changes in cardiometabolic risk factors associated with baseline and changes in BMI in the control group**

|  | Baseline BMI (Z-score) | | |  | Change in BMI (Z-score)* | | |
| --- | --- | --- | --- | --- | --- | --- | --- |
|  | Participants | Age- and sex-adjusted | Multivariable-adjusted^†^ |  | Participants | Age- and sex-adjusted | Multivariable-adjusted^†^ |
| Change in WC* | 2870 | 0.1924(0.1519,0.2329)^‡^ | 0.1811(0.1409,0.2214)^‡^ |  | 2858 | 0.4853(0.4570,0.5136)^‡^ | 0.5942(0.5671,0.6213)^‡^ |
| Change in PBF | 2805 | 0.2107(0.1733,0.2480)^‡^ | 0.2001(0.1615,0.2387)^‡^ |  | 2804 | 0.8326(0.7894,0.8757)^‡^ | 0.8453(0.8034,0.8871)^‡^ |
| Change in SBP | 2878 | 0.2699(0.2318,0.3080)^‡^ | 0.2677(0.2259,0.3095)^‡^ |  | 2864 | 0.2247(0.1574,0.2920)^‡^ | 0.2611(0.1952,0.3271)^‡^ |
| Change in DBP | 2878 | 0.1886(0.1515,0.2256)^‡^ | 0.1763(0.1357,0.2169)^‡^ |  | 2864 | 0.1139(0.0467,0.1811)^‡^ | 0.1347(0.0681,0.2014)^‡^ |
| Change in MAP | 2876 | 0.2384(0.2006,0.2763)^‡^ | 0.2293(0.1880,0.2706)^‡^ |  | 2862 | 0.1648(0.0971,0.2325)^‡^ | 0.1962(0.1296,0.2628)^‡^ |
| Change in TC | 2728 | -0.0183(-0.0450,0.0084) | -0.0207(-0.0504,0.0089) |  | 2691 | 0.0847(0.0348,0.1346)^‡^ | 0.0832(0.0328,0.1336)^‡^ |
| Change in HDL-C | 2726 | -0.1985(-0.2359,-0.1610)^‡^ | -0.1851(-0.2265,-0.1437)^‡^ |  | 2689 | -0.1977(-0.2675,-0.1280)^‡^ | -0.1898(-0.2589,-0.1207)^‡^ |
| Change in LDL-C | 2730 | 0.0024(-0.0272,0.0321) | -0.0163(-0.0486,0.0160) |  | 2693 | 0.1205(0.0655,0.1755)^‡^ | 0.1111(0.0565,0.1657)^‡^ |
| Change in log TG | 2733 | 0.2162(0.1838,0.2485)^‡^ | 0.2082(0.1724,0.2441)^‡^ |  | 2696 | 0.2969(0.2369,0.3568)^‡^ | 0.2994(0.2406,0.3582)^‡^ |
| Change in fasting glucose | 2731 | -0.0408(-0.0686,-0.0129)^‡^ | -0.0349(-0.0658,-0.0041)^‡^ |  | 2694 | 0.0151(-0.0374,0.0676) | 0.0128(-0.0402,0.0657) |
| Change in log insulin | 2466 | 0.3484(0.2831,0.4138)^‡^ | 0.3473(0.2771,0.4175)^‡^ |  | 2431 | 0.2843(0.1784,0.3902)^‡^ | 0.3310(0.2264,0.4356)^‡^ |
| Change in HOMA-IR | 2464 | 0.3186(0.2567,0.3805)^‡^ | 0.3192(0.2524,0.3860)^‡^ |  | 2429 | 0.2708(0.1690,0.3725)^‡^ | 0.3118(0.2111,0.4125)^‡^ |
| Change in CMRS | 2475 | 1.0579(0.9422,1.1736)^‡^ | 1.0003(0.8780,1.1227)^‡^ |  | 2467 | 1.0472(0.8702,1.2241)^‡^ | 1.2293(1.0623,1.3963)^‡^ |

BMI, body mass index; CMR, cardiometabolic risk; CRF, cardiorespiratory fitness; DBP, diastolic blood pressure; HDL-C, high-density lipoprotein cholesterol; HOMA-IR, homeostatic model assessment of insulin resistance; LDL-C, low-density lipoprotein cholesterol; MAP, mean arterial pressure; SBP, systolic blood pressure; TC, total cholesterol; TG, triglyceride.

*Changes in CMR factors were calculated by subtracting the results at baseline from those at follow-up.

^†^GLM was used to estimate multivariable-adjusted β and 95% CIs of cardiometabolic risk factors associated with percent body fat. Multivariable-adjusted analysis was adjusted for children within classes in school as clustering effects and characteristics of individuals including age, sex, corresponding CMR factor at baseline, puberty, grade, intervention, BMI, physical activity, energy intake, birthweight, household income, mother’s education, father’s education, mother’s BMI, and father’s BMI as fixed effects.

^‡^ Indicates significant associations. Benjamin-Hochberg procedure was used to control the false discovery rate at level 5% for multiple comparisons with the P-value cut-off point of significance was 0.0346, 0.0423, 0.0385, and 0.0423 for age- and sex-adjusted analysis (baseline PBF), multivariable-adjusted analysis (baseline PBF), age- and sex-adjusted analysis (change in PBF), and multivariable-adjusted analysis (change in PBF), respectively.

**Table S7. Changes in cardiometabolic risk factors associated with baseline and changes in PBF in the control group**

|  | Baseline PBF (Z-score) | | |  | Change in PBF (Z-score) | | |
| --- | --- | --- | --- | --- | --- | --- | --- |
|  | Participants | Age- and sex-adjusted | Multivariable-adjusted |  | Participants | Age- and sex-adjusted | Multivariable-adjusted |
| Change in BMI | 2856 | 0.0193(-0.0074,0.0459) | 0.0088(-0.0183,0.0359) |  | 2804 | 0.3790(0.3581,0.3998)^‡^ | 0.4255(0.4044,0.4467)^‡^ |
| Change in WC | 2845 | 0.0879(0.0640,0.1117)^‡^ | 0.0777(0.0536,0.1018)^‡^ |  | 2783 | 0.1905(0.1654,0.2157)^‡^ | 0.2587(0.2330,0.2844)^‡^ |
| Change in SBP | 2853 | 0.1887(0.1525,0.2249)^‡^ | 0.1822(0.1427,0.2216)^‡^ |  | 2790 | 0.0761(0.0223,0.1300)^‡^ | 0.1458(0.0907,0.2008)^‡^ |
| Change in DBP | 2853 | 0.1252(0.0893,0.1610)^‡^ | 0.1124(0.0735,0.1513)^‡^ |  | 2790 | 0.0707(0.0172,0.1241)^‡^ | 0.1139(0.0591,0.1687)^‡^ |
| Change in MAP | 2851 | 0.1605(0.1243,0.1968)^‡^ | 0.1497(0.1104,0.1890)^‡^ |  | 2788 | 0.0816(0.0277,0.1356)^‡^ | 0.1388(0.0837,0.1940)^‡^ |
| Change in TC | 2706 | 0.0175(-0.0084,0.0434) | 0.0185(-0.0098,0.0467) |  | 2623 | 0.0608(0.0221,0.0994)^‡^ | 0.0812(0.0411,0.1213)^‡^ |
| Change in HDL-C | 2704 | -0.1667(-0.2028,-0.1306)^‡^ | -0.1528(-0.1922,-0.1134)^‡^ |  | 2621 | -0.0220(-0.0759,0.0319) | -0.0713(-0.1267,-0.0159)^‡^ |
| Change in LDL-C | 2708 | 0.0153(-0.0137,0.0442) | -0.0069(-0.0380,0.0242) |  | 2625 | 0.0192(-0.0231,0.0615) | 0.0361(-0.0071,0.0793) |
| Change in log TG | 2711 | 0.1913(0.1601,0.2226)^‡^ | 0.1834(0.1493,0.2175)^‡^ |  | 2628 | 0.1135(0.0670,0.1599)^‡^ | 0.1885(0.1413,0.2356)^‡^ |
| Change in fasting glucose | 2709 | -0.0483(-0.0751,-0.0215)^‡^ | -0.0462(-0.0756,-0.0168)^‡^ |  | 2626 | -0.0475(-0.0876,-0.0075)^‡^ | -0.0609(-0.1025,-0.0193)^‡^ |
| Change in log insulin | 2448 | 0.2378(0.1792,0.2964)^‡^ | 0.2410(0.1784,0.3036)^‡^ |  | 2371 | 0.2154(0.1340,0.2968)^‡^ | 0.3214(0.2375,0.4052)^‡^ |
| Change in HOMA-IR | 2446 | 0.2195(0.1636,0.2753)^‡^ | 0.2224(0.1625,0.2823)^‡^ |  | 2369 | 0.1906(0.1122,0.2689)^‡^ | 0.2892(0.2083,0.3700)^‡^ |
| Change in CMRS | 2458 | 0.6168(0.5200,0.7135)^‡^ | 0.5660(0.4642,0.6677)^‡^ |  | 2414 | 0.2544(0.1227,0.3860)^‡^ | 0.5184(0.3850,0.6518)^‡^ |

BMI, body mass index; CMR, cardiometabolic risk; CRF, cardiorespiratory fitness; DBP, diastolic blood pressure; HDL-C, high-density lipoprotein cholesterol; HOMA-IR, homeostatic model assessment of insulin resistance; LDL-C, low-density lipoprotein cholesterol; MAP, mean arterial pressure; SBP, systolic blood pressure; TC, total cholesterol; TG, triglyceride.

*Changes in CMR factors were calculated by subtracting the results at baseline from those at follow-up.

^†^GLM was used to estimate multivariable-adjusted β and 95% CIs of cardiometabolic risk factors associated with percent body fat. Multivariable-adjusted analysis was adjusted for children within classes in school as clustering effects and characteristics of individuals including age, sex, corresponding CMR factor at baseline, puberty, grade, intervention, BMI, physical activity, energy intake, birthweight, household income, mother’s education, father’s education, mother’s BMI, and father’s BMI as fixed effects.

^‡^ Indicates significant associations. Benjamin-Hochberg procedure was used to control the false discovery rate at level 5% for multiple comparisons with the P-value cut-off point of significance was 0.0423, 0.0423, 0.0462, and 0.05 for age- and sex-adjusted analysis (baseline PBF), multivariable-adjusted analysis (baseline PBF), age- and sex-adjusted analysis (change in PBF), and multivariable-adjusted analysis (change in PBF), respectively.
